# A comprehensive review of natural compounds and their structure–activity relationship in Parkinson’s disease: exploring potential mechanisms

**DOI:** 10.1007/s00210-024-03462-4

**Published:** 2024-10-11

**Authors:** Rana M. Merghany, Salma A. El-Sawi, Asmaa F. Aboul Naser, Shahira M. Ezzat, Sherifa F. A. Moustafa, Meselhy R. Meselhy

**Affiliations:** 1 Department of Pharmacognosy, National Research Centre, 33 El-Buhouth Street, Cairo, 12622 Egypt; 2 Department of Therapeutic Chemistry, National Research Centre, 33 El Buhouth St, Cairo, 12622 Egypt; 3 Department of Pharmacognosy, Faculty of Pharmacy, Cairo University, Kasr El-Aini Street, Cairo, 11562 Egypt; 4 Department of Pharmacognosy, Faculty of Pharmacy, October University for Modern Sciences and Arts (MSA), Giza, 12451 Egypt

**Keywords:** Parkinson’s disease, Natural compounds, Flavonoids, Inflammation, MAO-B, α-Synuclein

## Abstract

Parkinson’s disease (PD) is a neurodegenerative disorder characterized by the progressive loss of dopamine-producing cells in the Substantia nigra region of the brain. Complementary and alternative medicine approaches have been utilized as adjuncts to conventional therapies for managing the symptoms and progression of PD. Natural compounds have gained attention for their potential neuroprotective effects and ability to target various pathways involved in the pathogenesis of PD. This comprehensive review aims to provide an in-depth analysis of the molecular targets and mechanisms of natural compounds in various experimental models of PD. This review will also explore the structure–activity relationship (SAR) of these compounds and assess the clinical studies investigating the impact of these natural compounds on individuals with PD. The insights shared in this review have the potential to pave the way for the development of innovative therapeutic strategies and interventions for PD.

## Introduction

Parkinson’s disease (PD) is ranked as the second most common neurodegenerative disorder worldwide, and its prevalence has been steadily rising, yet a cure remains elusive (Abubakar et al. 2015). PD affects approximately 1–2% of individuals over the age of 60 (Klemann, et al. 2017). The number of PD cases ranges from 5 to 35 per 100,000 individuals, with an increased frequency observed in older age groups. The prevalence of PD is projected to double by the year 2030, indicating a substantial rise in the disease’s occurrence (Aarsland et al. 2021). PD is characterized by the progressive deterioration of dopaminergic neurons’ structure and function, leading to muscle weakness and impairment of the body’s normal functions. Various genetic, biochemical, molecular, and environmental factors contribute to the progression of PD, leading to cell death, due to triggering cellular apoptosis signaling (Trist et al. 2019).

In recent years, there has been increased interest in the use of plant-derived compounds, for the management of PD (Balakrishnan et al. 2021). These natural compounds are believed to have fewer side effects compared to synthetic compounds (Jung and Kim 2018). The recent advances made in managing PD hold the promise of effectively controlling the disease, alleviating its symptoms, and enhancing the overall wellbeing and quality of life of PD patients. Phytochemicals encompass a wide range of chemical, biochemical and molecular properties, that have attracted significant interest as potential candidates for managing PD. Several natural compounds have demonstrated the potential in modulating multiple signaling pathways, neurotransmitters and neurotrophic factors, inhibiting α-synuclein (α-Syn) aggregation and fibrillation, protecting mitochondria and acting as antioxidants and anti-inflammatory agents (Shahpiri et al. 2016).

This detailed review focuses on the impact of natural compounds on PD with their structure–activity relationship (SAR), as discussed in the literature. It also highlights the targets involved in the degenerative processes of the disease Also, the review presents the findings obtained from preclinical and clinical studies about the therapeutic potential of these natural compounds.

## Method

A comprehensive search in the literature was done using the PubMed, Web of Science, and Pubchem databases via Google search. Only topics that yielded meaningful results relevant to the management of PD were included in this review. The topics covered a range of elements including “Parkinson’s disease (PD),” “pathophysiology,” “oxidative stress in PD development,” “available natural compounds for the management of PD,” “preclinical,” and “clinical.” Additionally, topics related to specific classes of compounds, including “flavonoids,” “alkaloids,” “saponins,” “terpenes,” “coumarins,” “tannins,” and “essential oil” as well as “SAR” were included.

### Overview of Parkinson’s disease

#### Symptoms

PD is characterized by the progressive degeneration of dopaminergic neurons, resulting in the depletion of dopamine (DA) in the striatum and the formation of Lewy bodies in the Substantia nigra (SN). These neuropathological changes are closely associated with motor symptoms such as resting tremors, rigidity, bradykinesia, gait difficulties, postural instability, and behavioral problems. Non-motor symptoms, including depression, anxiety, emotional changes, cognitive impairment, sleep disturbances, and olfactory dysfunction, are also common in PD (Bloem et al. 2021).

#### Etiology

Various factors contribute to the increase in the burden of PD, including the risk factors (diabetes and obesity), genetic mutations, increasing smoking rates, and the growing impact of industrial and pesticide pollution. The combination of high disease prevalence, demographic changes, industrial pollution, and excessive pesticide use (rotenone and paraquat) has led to a global Parkinson’s pandemic as well as placing a financial load on the healthcare system, society, the rate of output, and the quality of life of the patients. This requires focused planning and innovative approaches for PD management (Dorsey et al. 2018).

In relation to genetic factors, only a handful of specific genes have been definitively linked to monogenic forms of the disease such as SNCA, LRRK2, and VPS35 for autosomal-dominant PD, and PRKN, PINK1, and PARK7 for autosomal-recessive forms (Bandres-Ciga et al. 2020). Additionally, mutations in GBA1 have emerged as the most significant genetic risk factor for PD (Bandres-Ciga et al. 2020).

For autosomal-dominant PD, VPS35 was initially discovered through whole exome sequencing as a causative element in late-onset PD. Subsequent research unveiled the VPS35 p.D620N mutation to be classified as pathogenic. Penetrance studies concerning the VPS35 p.D620N mutation indicate that 25% of carriers exhibit PD symptoms by the age of 45 or younger, with a median onset age of 49 years, and 75% of individuals showing symptoms by age 59 or older. The typical phenotype associated with this variant often presents classic PD symptoms and a strong response to L-dopa. However, it is characterized by fewer cognitive and neuropsychiatric features and hyposmia in around 50% of patients (Khani et al. 2024).

As well, mutation in the LRRK2 gene has been associated with familial PD. Statistical data shows that 15–20% of PD patients have family members with PD symptoms, and relatives of a person with PD patient have a 3–4 times higher risk of developing PD compared to the general population (Vijayakumar et al. 2016). Also, mutations in the SNCA gene, which plays a role in synaptic function and DA transmission, are associated with the formation of Lewy vesicles, impaired mitochondrial function, age-related neurodegeneration, and death of dopaminergic neurons.

Similarly, autosomal-recessive PD, primarily linked to mutations in the PRKN, PINK1, and PARK7 genes, exhibits distinctive clinical characteristics. The clinical profile associated with mutations in PRKN, PINK1, and PARK7 typically includes classic motor symptoms of Parkinsonism that appear at a younger age, typically before or around 30 years, and are linked with a slower disease progression. Common features, regardless of genetic background, encompass dyskinesia and dystonia, which are notably prevalent in cases of early-onset PD. In comparison to idiopathic PD, non-motor symptoms are less common, particularly in instances involving PRKN mutations, and neuropathological assessments often reveal a lower presence or near absence of Lewy bodies (Doherty et al. 2013). A systematic review indicated varying degrees of cognitive impairment connected with these mutations. Cognitive issues were identified in less than 10% of PRKN cases, while PARK7 and PINK1 cases displayed rates around 29% and 58%, respectively. Nonetheless, the completeness of comprehensive clinical data in reports concerning these mutations remains largely inadequate. As highlighted in the MDSGene Systematic Review by Kasten et al. (2018)*,* there exists a notable amount of missing phenotypic data across publications, encompassing both non-motor symptoms and key motor features (Kasten et al. 2018). On the other hand, the inactivation of PRKN leads to the accumulation of the PRKN-interacting substrate (PARIS). This accumulation contributes to PD by suppressing the activity of peroxisome proliferator-activated receptor-gamma coactivator-1 alpha (PGC-1α), a transcriptional coactivator involved in regulating mitochondrial biogenesis and oxidative metabolism (Shin et al. 2011). PD is also linked to mutations in DJ-1 gene that contribute to oxidative stress, mitochondrial dysfunction, and neurodegeneration (Domingo and Klein 2018).

On the other hand, mutations in one of the two copies of the GBA1 gene, typically linked with Gaucher’s disease (GD) when occurring in both copies, have emerged as a significant risk factor for PD and Lewy body dementia. These harmful variations disrupt the function of the glucocerebrosidase enzyme, leading to reduced enzymatic activity and potentially increasing the aggregation of α-Syn (Sidransky et al. 2009). The array of GBA1 mutations seen in various populations also varies significantly, with specific mutations carrying different levels of risk. For instance, the p.N409S mutation, commonly found in European and Ashkenazi Jewish populations, is linked to a less severe PD phenotype, while the p.L483P mutation is associated with a more aggressive form of PD. Both mutations lead to GD when present in a homozygous state (Smith and Schapira 2022). Additionally, gene mutations associated with early-onset PD have been identified, including the GSK3β gene that is involved in neuronal function and survival (Golpich et al. 2015).

On the other hand, environmental factors including the excessive use of pesticides can lead to neuronal damage that occurs in PD due to the presence of oxidative stress caused by circulating free radicals in the brain. Oxidative stress, a hallmark feature of PD, results from the imbalance between reactive oxygen species production and antioxidant defense mechanisms, leading to cellular damage and neurodegeneration (Taylor et al. 2013). This can result in decreased glutathione levels in the SN relative to other brain regions, continual formation of reactive oxygen species by autoxidation, breakdown of DA by the monoamine oxidase B (MAO-B) enzyme, and loss of cholinergic function (Kumar et al. 2018). Along with oxidative stress, neuroinflammation and systemic inflammation are intricately intertwined processes that significantly contribute to disease progression. Neuroinflammation, characterized by microglial activation and the release of pro-inflammatory cytokines, plays a central role in the degeneration of dopaminergic neurons in the SN. Various studies have implicated neuroinflammation and cytotoxic factors such as interleukins (ILs), nitric oxide (NO), reactive oxygen species (ROS), and tumor necrosis factor-α (TNF-α) in the neurodegenerative processes underlying PD (Joshi and Singh 2018). Systemic inflammation, often observed in PD patients, can exacerbate neuroinflammation through the infiltration of peripheral immune cells into the brain, further amplifying the inflammatory response (Li et al. 2024). Additionally, Lewy bodies formed in neurons are intimately linked to α-Syn aggregation and fibrillation, which may play a role in the etiology of PD (Dettmer et al. 2016). The cause of the death of neuronal cells is yet unknown; however, dopaminergic cell death has been linked to oxidative stress, subsequent apoptotic cell death pathways, and malfunction of the mitochondria that are important for energy metabolism and neurotransmission (Franco-Iborra et al. 2016).

#### Experimental models

The majority of our understanding of PD development originates from studies conducted in experimental models of PD, particularly those induced by neurotoxins (Bové et al. 2005). 6-hydroxydopamine (6-OHDA) is a commonly used neurotoxin that resembles catecholamines and is recognized by DA and NE transporters. Once inside the cell, 6-OHDA undergoes oxidation, leading to the production of reactive oxygen species and mitochondrial dysfunction, ultimately causing the death of dopaminergic neurons. The selective toxicity of 6-OHDA to catecholaminergic neurons makes it an accurate and effective model for PD (Blum et al. 2001). Another neurotoxin, 1-methyl-4-phenyl-1,2,3,6-tetrahydropyridine (MPTP), is structurally similar to environmental toxins and is metabolized to MPP + by the enzyme MAO-B. MPP + enters dopaminergic neurons via the DA transporter and inhibits mitochondrial complex I, resulting in decreased ATP levels, the subsequent apoptosis, and necrosis of dopaminergic neurons (Speciale 2002). Rotenone once used as a pesticide, also inhibits mitochondrial complex I activity, generates reactive oxygen species, and selectively induces the death of dopaminergic neurons (Lawana and Cannon 2020). Paraquat (another pesticide), structurally similar to MPTP, enters dopaminergic neurons via the DA transporter and induces oxidative stress responses and apoptosis (Colle and Farina 2021). Other pesticides such as Dichlorodiphenyltrichloroethane (DDT) and dieldrin can also be used to induce PD (Hatcher et al. 2008). As well, lipopolysaccharide (LPS), a component of the outer membrane of certain bacteria, can induce an immune response and neuroinflammation for PD (Tufekci et al. 2011). These neurotoxic models help simulate PD symptoms and facilitate drug screening and therapeutic development (Thirugnanam and Santhakumar 2022). On the other hand, Reserpine (a natural alkaloid) is known for its irreversible inhibition of the vesicular monoamine transporter 2 (VMAT-2) and is used to induce PD-like symptoms in rats by depleting monoamines and affecting locomotor activities (Li et al. 2022).

In addition to neurotoxin-induced models, genetic models of PD have been developed by introducing mutations into genes associated with PD, such as SNCA, LRRK2, PRKN, PINK1, and DJ-1 (Domingo and Klein 2018). Researchers employ a variety of techniques to create and analyze transgenic PD models in animals (Pan et al. 2024). For instance, viral vector delivery methods involve using adeno-associated viruses or lentiviruses to transport specific genes or gene-editing tools into the animal brain (Ye, et al. 2024). In addition, CRISPR/Cas9 gene editing enables precise mutations associated with PD to be introduced into the animal genome (Mathur and Seamon 2024). Furthermore, gene transfer techniques such as electroporation or stereotaxic injection are utilized to introduce transgenes into specific brain regions of the animal (Heller and Hamilton 2024).

To better emulate human PD and address research needs, it has been noted that neurotoxins have a significant impact on transgenic animals compared to their non-modified counterparts (He, et al. 2024). For example, when DJ-1 transgenic mice underwent modeling through a combination of DJ-1 overexpression and MPTP neurotoxin administration, the resulting mice displayed more severe degeneration of DA neurons and increased cell death compared to using a single method alone (Heinemann et al. 2016). Additionally, extended and chronic rotenone injections in LRRK2 transgenic mice lead to DA neuron degeneration in the SN pars compacta and striatum, along with α-syn aggregation and the development of PD-associated dyskinesia (Ng et al. 2009).

#### Current managements

Unfortunately, PD is a chronic condition that progresses gradually and currently has no known cure. However, PD management involves a multifaceted approach encompassing pharmacological and non-pharmacological treatments (Muleiro Alvarez et al. 2024). In the context of pharmacological treatments, it is important to note that medications used may have significant side effects. As well, long-term of its use may lead to decreased effectiveness over time (Muleiro Alvarez et al. 2024). L-dopa remains a cornerstone medication for addressing motor symptoms, although it can lead to dyskinesia and motor fluctuations over time (Booth 2024). Dopamine agonists and MAO-B inhibitors mimic dopamine’s effects but may result in side effects like nausea and hallucinations (Goldenberg 2008). Catechol-o-methyl-transferase (COMT) inhibitors can extend L-dopa’s efficacy but may cause diarrhea and dyskinesia. Anticholinergics help with tremors but can lead to dry mouth and cognitive issues (Marsili et al. 2017; Höglinger and Trenkwalder 2024). On the other hand, non-pharmacological interventions like physical therapy improve mobility with potential muscle soreness, while speech and occupational therapy target speech and daily living challenges with minimal adverse effects. Deep Brain Stimulation (DBS) surgically implants electrodes for symptom management, with risks including infection and hardware-related issues (Nemade et al. 2021; Hartmann-Nardin, et al. 2024).

Despite these challenges, These treatments remain a valuable approach in the management of PD, helping individuals cope with the disease and alleviate symptoms (So et al. 2024). Consequently, there is a need to explore new, cheap, and safe natural neuroprotective agents with minimal side effects.

### Mechanisms of action of natural compounds

**Fig. 1:**
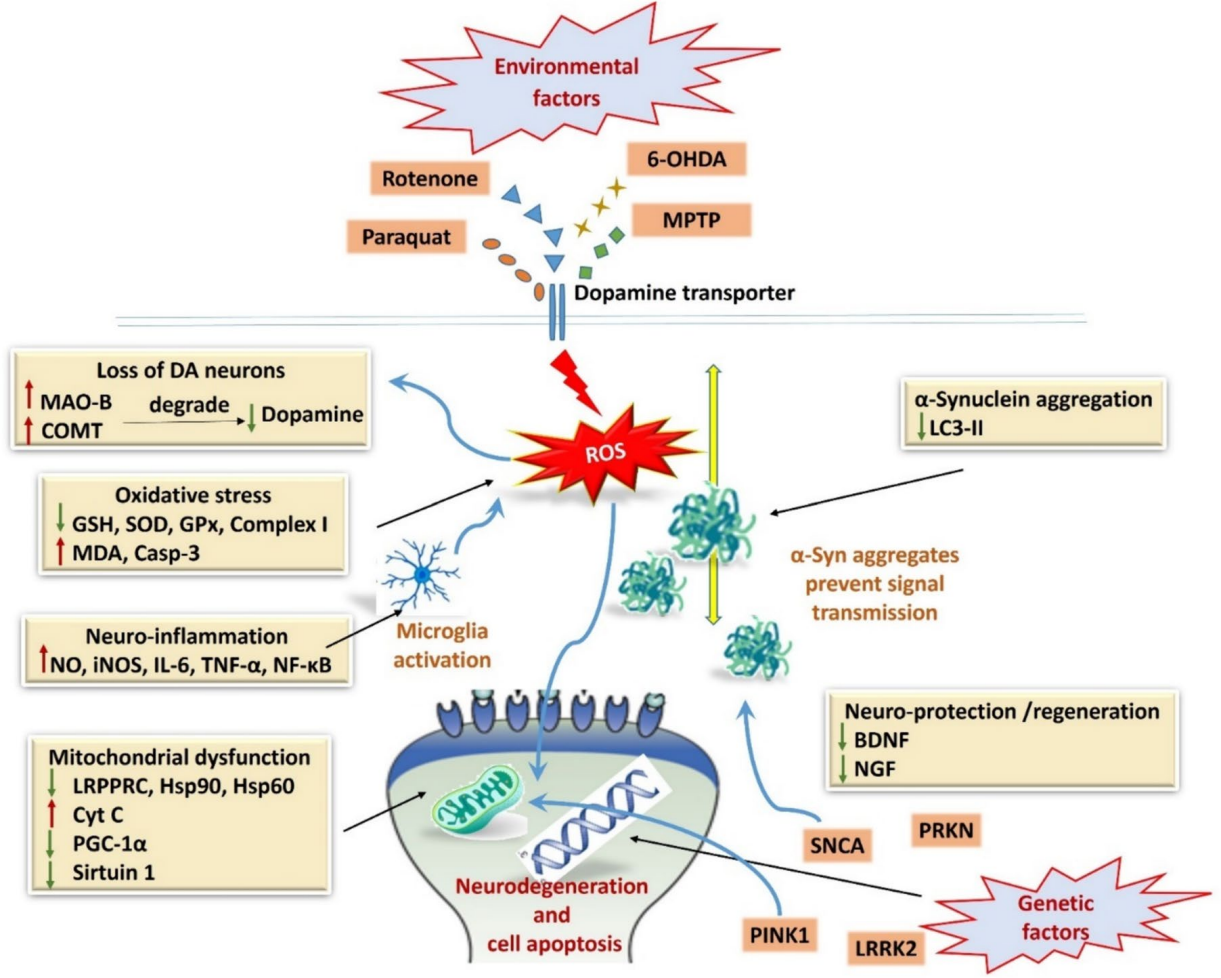
Proposed physiological mechanisms associated with the development of Parkinson’s disease (PD). Red up-arrow indicates an increase in the level, activity, or expression. Green down-arrow indicates a decrease in the level, activity, or expression

PD encompasses a complex interplay of diverse and intricate physiological mechanisms **(**Fig. 1**)**. Natural compounds could target the involved pathways through different mechanisms of action.

#### Modulation of neurotransmitters

Natural compounds can influence the levels and activity of neurotransmitters in the brain, which are essential for proper neuronal communication. For instance, some compounds can increase the effect (agonist), synthesis (tyrosine hydroxylase (TH) activator), or release (MAO-B inhibitor) of DA, the main neurotransmitter that is depleted in PD and responsible for its motor symptoms, particularly in the SN region (Alosaimi et al. 2022). MAO is a group of enzymes involved in the metabolism of neurotransmitters such as DA, serotonin (5-HT), and norepinephrine (NE). In PD, the two forms of MAO, MAO-A, and MAO-B, have been implicated in the pathogenesis of the condition. MAO-A is primarily responsible for the breakdown of 5-HT and NE and plays an important role in psychiatric conditions such as depression associated with PD. MAO-B is primarily involved in the breakdown of DA and is implicated in the neurological disorders of PD. In PD, the inhibition of MAOs is one of the neuroprotective approaches used in the management of the disease (Tan et al. 2022). In addition, TH plays a crucial role in the biosynthesis of DA by catalyzing the formation of L-dopa. PD can be considered a deficiency syndrome of TH in the striatum (Nagatsu et al. 2019). On the other hand, COMT inhibitors can prevent the degradation of L-dopa (Gershanik 2015). Additionally, the adenosine A2A receptor is a type of G protein-coupled receptor (GPCR) that is predominantly expressed in the brain, particularly in areas involved in motor control, such as the basal ganglia. Studies have shown that the adenosine A2A receptor interacts with other receptors, such as DA D2 receptors, in a complex manner in the basal ganglia. Dysfunction in these interactions, specifically the overactivation of adenosine A2A receptors, can lead to an imbalance in the basal ganglia circuitry, resulting in abnormal motor function and contributing to the motor symptoms characteristic of PD. Based on this understanding, inhibiting the adenosine A2A receptor by using antagonists, aims to restore the balance between adenosine A2A and DA D2 receptor signaling in the basal ganglia, potentially alleviating motor symptoms (Pinna 2014).

#### α-Synuclein aggregation and fibrillation inhibition

α-Syn is a protein that forms abnormal aggregates and fibrils in PD. Natural compounds can show inhibitory effects on α-Syn aggregation and fibrillation. By interfering with the formation of toxic protein aggregates, these compounds may help protect neurons from damage and slow down disease progression (Fields et al. 2019). As well, natural compounds can increase the level of Microtubule-associated protein 1A/1B-light chain 3 (LC3-II), a marker of autophagy, that is decreased in PD which leads to the accumulation of α-Syn aggregates (Wang et al. 2017a). Recently, studies have found that disruption of the MALAT1/miR-129/SNCA pathway has been implicated in PD and can influence α-Syn expression and aggregation (Abrishamdar et al. 2022).

#### Mitochondrial protection and energy regulation

Natural compounds can protect mitochondria, the cellular powerhouses responsible for energy production, from oxidative damage. They can enhance mitochondrial function, improve energy metabolism, and maintain mitochondrial integrity. By preserving mitochondrial function, natural compounds help maintain optimal neuronal energy supply and reduce cellular stress by balancing the activities of LDH (Lactate dehydrogenase) and SDH (Succinate dehydrogenase) enzymes that are involved in cellular metabolism and are often used as markers in various diagnostic tests and research studies to assess tissue damage (Grünewald et al. 2019). As well, cytochrome c (Cyt C), a protein involved in the electron transport chain of mitochondria, is released from damaged mitochondria and can trigger apoptotic pathways, contributing to the degeneration of dopaminergic neurons (Meng et al. 2017). Additionally, PGC-1α, an integral for the regulation of mitochondrial biogenesis and oxidative metabolism, plays a crucial role in maintaining cellular energy homeostasis (Scarpulla 2011). Studies have shown that PGC-1α expression is reduced in PD, which may contribute to mitochondrial dysfunction and compromised energy production in dopaminergic neurons (Bennett et al. 2022). In addition, Sirtuin 1, a protein encoded by the SIRT1 gene, is involved in regulating cellular processes such as DNA repair, apoptosis, and mitochondrial function, where its levels are reduced in PD (Li et al. 2020a). Other factors such as LRPPRC (Leucine-rich pentatricopeptide repeat-containing protein), Hsp90 (Heat shock protein 90), and Hsp60 (Heat shock protein 60) are involved in mitochondrial function and maintenance, where their levels decrease in PD (Han et al. 2014).

#### The regulation of endocannabinoid and the cholinergic systems

In PD, there is evidence of dysfunction in both the endocannabinoid system and the cholinergic system. These systems have been studied for their potential roles in the pathophysiology and control of PD (Scherma et al. 2016). The endocannabinoid system (ECS) consists of cannabinoid receptors (CB1 and CB2), endocannabinoids (such as anandamide and 2-AG), and enzymes involved in their synthesis and degradation. Phytocannabinoids proved to reverse the alterations observed in the ECS, including changes in cannabinoid receptor expression and dysregulation of endocannabinoid signaling due to Parkinsonism. Activation of cannabinoid receptors has been reported to have neuroprotective effects, reducing inflammation, oxidative stress, and excitotoxicity in preclinical models of PD (Lutz 2022).

The cholinergic system, particularly the nicotinic acetylcholine receptors (nAChRs), plays a crucial role in motor control and cognition. In PD, there is a loss of cholinergic neurons in the basal forebrain and a reduction in nicotinic receptor availability. Nicotine, a selective agonist of nAChRs, has been studied for its potential neuroprotective effects in PD. Nicotine can modulate DA release, enhance attention and cognitive function, and improve motor symptoms (Quik et al. 2015).

#### Antioxidant activity

Natural compounds possess antioxidant properties by neutralizing harmful free radicals and reducing oxidative stress by improving some biomarkers such as glutathione (GSH), superoxide dismutase (SOD), glutathione peroxidase (GPx), and complex I as well as decreasing the level of malondialdehyde (MDA) and the activity of caspase-3 (Casp-3) (Essa et al. 2014). GSH is an important antioxidant molecule that helps protect cells from oxidative damage. Studies have shown a decrease in GSH concentration in PD, suggesting a disturbance in the antioxidant defense system, where GCLC (glutamate-cysteine ligase catalytic subunit), GCLM (glutamate-cysteine ligase modifier subunit), and GSR (glutathione reductase) are important enzymes involved in the synthesis and recycling of GSH (Wu et al. 2015). SOD is another enzyme that helps neutralize superoxide radicals. As well, GPx1 is an enzyme involved in the detoxification of hydrogen peroxide and lipid peroxides through the reduction of glutathione (Ruszkiewicz and Albrecht 2015). Complex I is a part of the mitochondrial electron transport chain, and its inhibition leads to increased oxidative stress and further damage to neurons. Excessive increased lipid peroxidation (LPO), which is the oxidation of lipids in cell membranes, and its product MDA have been reported in PD. These processes contribute to oxidative stress and neuronal death. As well, Casp-3 serves as a central point in both mitochondria-dependent and mitochondria-independent apoptotic pathways. When Casp-3 is activated, it acts on various proteins, including Poly (ADP-ribose) polymerase (PARP), which plays a crucial role in repairing damaged DNA (Lu et al. 2017). As a result, Casp-3 promotes apoptosis and its inhibition by natural compounds is a promising target. In addition, natural compounds can increase the level of nuclear factor erythroid 2-related factor 2 (Nrf2), a transcription factor that regulates the expression of antioxidant and cytoprotective genes through the antioxidant response element (ARE), where their levels are decreased in PD (Aguiar et al. 2016). On the other hand, Keap1 (Kelch-like ECH-associated protein 1) is a protein involved in the regulation of cellular antioxidant defense mechanisms. It plays a crucial role in controlling the activity of Nrf2. In PD, dysregulation of the Keap1/Nrf2 pathway has been reported, leading to impaired antioxidant defenses and increased vulnerability to oxidative stress (Mahapatra 2018).

#### Anti-inflammatory properties

Concerning the anti-inflammatory activity, natural compounds can inhibit the production of NO and the activity of nitric oxide synthase (iNOS), which play a role in various physiological processes, including neurotransmission and immune defense. As well, pro-inflammatory cytokines such as IL-6 and TNF-α are elevated in PD. They can induce glial cell activation, increase reactive oxygen species production, and contribute to apoptosis (Kempuraj et al. 2021). Another novel inflammatory cytokine is IL-33 which exerts its effects by binding to the orphan receptor suppression of tumorigenicity 2 (ST2), leading to the activation of various signaling pathways resulting in neuronal death (Sun et al. 2021). Additionally, NLRP3 (NOD-like receptor family, pyrin domain-containing 3), an inflammasome protein complex involved in the activation of inflammatory responses is activated in PD leading to the release of pro-inflammatory cytokines, including IL-1β, which may contribute to neuronal damage and progression of the disease (Khot, et al. 2022). As well, microglia (the immune cells of the central nervous system) are activated in PD and can release inflammatory molecules, contributing to neuroinflammation and neuronal damage (Ramirez et al. 2017). PD is characterized by chronic neuroinflammation, often accompanied by elevated levels of C-reactive protein (CRP) and Cox-2 (an enzyme involved in the production of prostaglandins, including PGE2), along with other inflammatory mediators such as ILs, TNF-α, and INF-γ (Mehta et al. 2023; Fritz et al. 2016). In addition, some other important molecular factors and pathways represent a complex network of interactions with PD pathogenesis. For instance, matrix metalloproteinases (MMP) are enzymes involved in the breakdown of extracellular matrix components. Increased MMP activity has been observed in PD, suggesting a potential role in disease progression. MMPs are thought to contribute to neuroinflammation, oxidative stress, and the disruption of blood–brain barrier integrity, which are all implicated in PD pathophysiology (Behl et al. 2021). As well, JNK and p38 are members of the mitogen-activated protein kinase (MAPK) family. Activation of JNK and p38 has been linked to neuronal cell death and inflammation in PD. These kinases are involved in intracellular signaling cascades that regulate cellular responses to stress, and their dysregulation can contribute to the degeneration of dopaminergic neurons (Jha et al. 2015). Additionally, JAK2 (Janus kinase 2) and STAT3 (signal transducer and activator of transcription 3) are signaling molecules involved in various cellular processes, including inflammation and immune responses. In PD, there is evidence of dysregulated JAK2/STAT3 signaling. Abnormal phosphorylation of JAK2 and STAT3 has been observed in the brains of individuals with PD, suggesting their involvement in the inflammatory processes and neurodegeneration associated with the condition (Lashgari et al. 2021). On the other hand, p53 is a transcription factor known for its role in regulating cell cycle progression, DNA repair, and apoptosis. Studies have suggested that p53 activation may contribute to the neurodegenerative processes in PD and it can mediate cell death pathways and oxidative stress in dopaminergic neurons (Luo, et al. 2022). As well, Bax and Bcl-2 are proteins involved in the regulation of apoptosis Imbalances between increased pro-apoptotic Bax and decreased anti-apoptotic Bcl-2 have been implicated in PD (Liu et al. 2018). Additionally, intracellular calcium dysregulation is implicated in neuronal cell death. Calbindin D28K is a calcium-binding protein involved in the regulation of calcium homeostasis in neurons, which is reduced in PD (Ricke et al. 2020).

#### Neuroprotection and neuroregeneration

Natural compounds can exert direct neuroprotective effects by preventing neuronal cell death and promoting cell survival. They can inhibit apoptosis, reduce neuronal damage caused by oxidative stress and inflammation, and enhance cellular defense mechanisms. Additionally, natural compounds can promote the production of neurotrophic factors, such as brain-derived neurotrophic factor (BDNF) and nerve growth factor (NGF), which support the survival and growth of neurons. These effects can enhance neuronal function, promote neuroplasticity, and potentially protect against neurodegeneration (Solayman et al. 2017).

#### Attenuation of behavioral impairments and cognitive deficits

PD is a complex neurodegenerative disorder characterized not only by motor symptoms but also by a range of behavioral impairments and cognitive deficits that significantly impact patients’ quality of life (Kalaba and Güzeloğlu 2024). Behavioral symptoms such as depression, anxiety, apathy, and impulse control disorders, along with cognitive impairments including executive dysfunction, attention deficits, and memory issues, pose considerable challenges for individuals with PD (Maristany, et al. 2024). Several natural compounds have shown promise in addressing these deficits. Curcumin, a compound found in turmeric, has demonstrated anti-inflammatory and antioxidant properties that may help alleviate cognitive impairments in PD by targeting neuroinflammation and oxidative stress (Turer and Sanlier 2024). Green tea catechins, specifically epigallocatechin gallate (EGCG), have been linked to improvements in cognitive function and behavioral symptoms in PD due to their neuroprotective effects (Pandit et al. 2024). Omega-3 fatty acids from sources like fish oil have anti-inflammatory properties that could benefit cognitive function and mood regulation in PD patients (Rao et al. 2024). Resveratrol, found in red grapes and berries, has antioxidant and anti-inflammatory effects that may help mitigate cognitive decline and improve behavioral symptoms in PD (Jadidian et al. 2024). As well, *Ginkgo biloba* and its main active ginkgolides are known for their cognitive-enhancing effects (Ali et al. 2024).

## Results

### Natural compounds and Parkinson’s disease

The search for effective beneficial interventions for PD has led to extensive research on natural compounds derived from various sources. This review focuses on preclinical (in vitro and in vivo) and clinical studies conducted to investigate the potential neuroprotective effects of natural compounds (non-volatile and volatile constituents) in PD.

#### Non-volatile compounds

##### Preclinical studies

In vitro studies such as establishing cell culture models, assessing cellular viability and apoptosis, as well as investigating the modulation of signaling pathways, are preliminary steps for the in vivo studies that represent animal models of PD, including toxin-induced models (e.g., rotenone) and genetic models (e.g., SNCA transgenic mice). Where behavioral assessments such as wire-hanging and open-field tests are used to evaluate motor functions. In addition, the changes in different biomarkers are assessed (Vijayakumar et al. 2016).

**Fig. 2:**
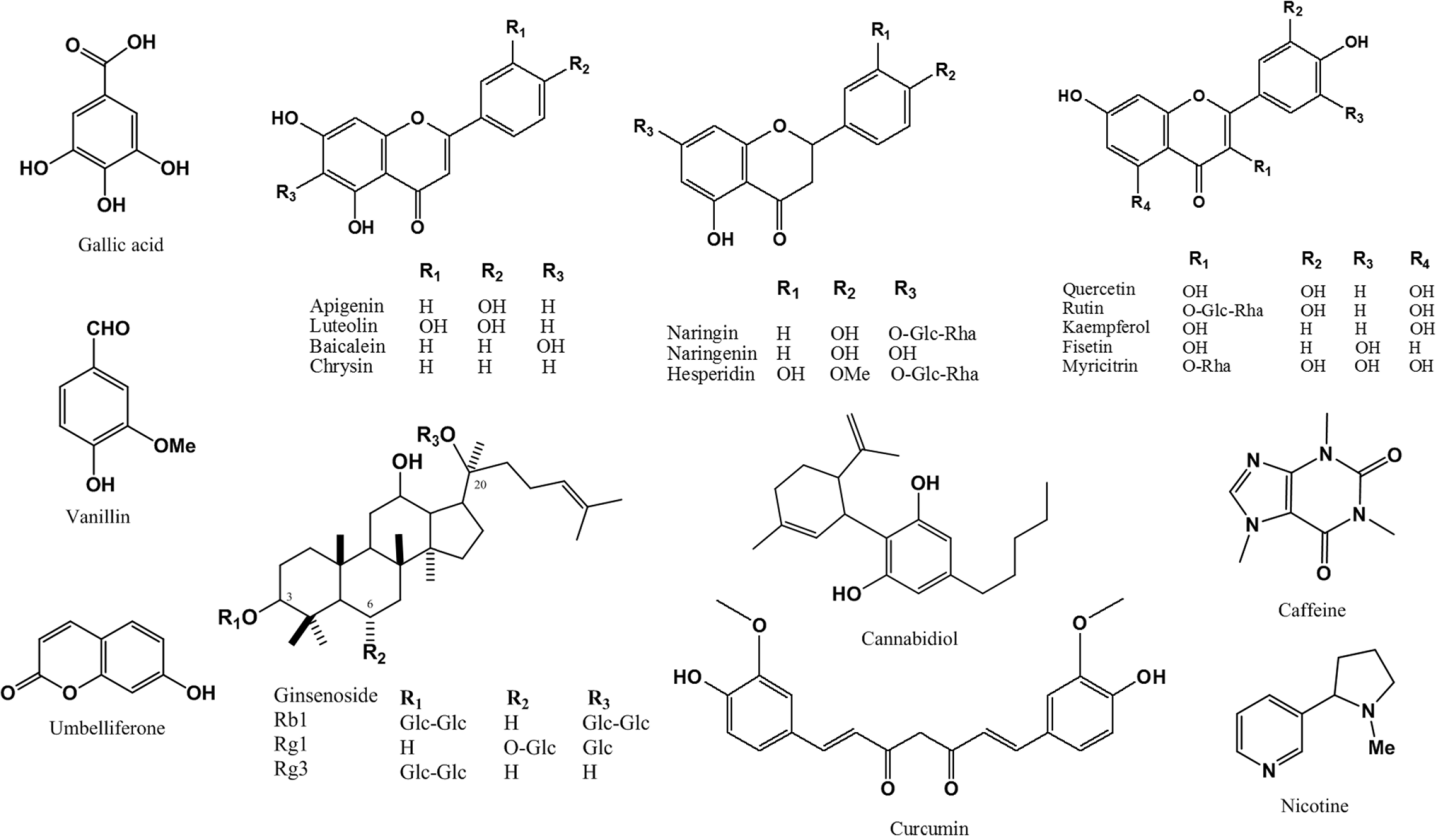
Chemical structures of some bioactive compounds that have been reviewed for their anti-PD effects

The natural compounds are categorized based on their chemical class, offering a valuable reference for current advancements in the study of natural compounds for PD management **(**Table 1 and Fig. 2).

**Table 1:** Preclinical evidence on the therapeutic efficacy of different natural compounds against Parkinson’s disease

Compound name	Type of study	Type of model	Dose/concentration	Mechanisms	Reference
**Terpenoids**					
Astragaloside IV	In vitro	6-OHDA-induced neurotoxicity in SH-SY5Y cells	25, 50, 100, 150, or 200 µM	Decrease in IL-1β, IL-6 and TNF-α. Decrease in MDA and ROS. Increase in SOD. Regulation of Bax/Bcl-2 ratio. Conserved the functional integrity of the mitochondria. Increase in phosphorylation of JAK2 and STAT3	Xu et al. 2021)
Asiatic Acid	In vitro	MPTP-induced neurotoxicity in SH-SY5Y cells	0.1, 1.0, or 10 nM	Decrease in ROS. Inhibition of NLRP3 inflammatory vesicle	Chen et al. 2019)
	In vivo	MPTP-induced PD in mice	20, 40, or 80 mg/kg	Decrease in NO, IL-1β, IL-6, and TNF-α. Inhibition in TLR2 expression. Increase in GSH	Chao et al. 2016)
α-Tocotrienol	In vivo	6-OHDA-induced PD in rats	10 mg/kg	Decrease in neuronal degradation and inflammatory mediators. Increase in DA neurons	Kumari et al. 2021)
Carnosic acid	In vitro	6-OHDA-induced neurotoxicity in SH-SY5Y cells	5 µM	Increase GCLC, GCLM, GSR, and SOD. Regulation of Bcl-2/Bax. Decrease in cleaved PARP	Wu et al. 2015)
	In vivo	6-OHDA-induced PD in rats	20 mg/kg	Improvement in the locomotor activity. Decrease in LPO and increase in SOD. Regulation of Bcl-2/Bax	Wu et al. 2015)
Ginkgolide B	In vitro	Rotenone-induced neurotoxicity in PC12 cells	Combination of Ginkgolide B (25 µM) and Protocatechuic acid (0.6 mM)	Increase in cell viability. Decrease in ROS and cell apoptosis. The effects were better than using Ginkgolide B alone	Wu et al. 2020)
		α-Syn-induced neurotoxicity in SH-SY5Y cells	Ginkgolide B or Bilobalide 1, 5, 10, or 50 µM	Reduction in cell apoptosis and aggregated α-Syn	Hua et al. 2017)
	In vivo	MPTP-induced PD in mice	Ginkgolide B (20 mg/kg) combined with Protocatechuic acid (5 mg/kg)	Improvement in motor ability. Reduction in the injury of neurons. Improvement in antioxidant enzymes and TH^+^ neurons number	Wu et al. 2020)
Ginkgolide K	In vivo	MPTP-induced PD in mice	20 mg/kg	Enhancement of motion function. Decrease in DA neuron loss. Immunomodulation by inhibiting CD4^+^IFN-γ^+^, α-Syn specific autoantibodies, and microglia activation. Increase the expression of BDNF and GDNF	Miao et al. 2022)
Ginsenoside Rb1 or Rg1 or Rg3	In vitro	α-Syn-induced neurotoxicity in-BE(2)-M17 cells	25 µM	Increase in cell viability. Decrease in α-Syn fibrillation and increase in α-Syn digestion	Ardah et al. 2015)
Oleuropein	In vitro	6-OHDA-induced neurotoxicity in PC12 cells	20 or 25 µg/mL	Increase in cell viability. Decrease in Casp-3, ROS, and DNA fragmentation. Regulation of Bax/Bcl-2	Pasban-Aliabadi, et al. 2013)
Tanshinone I	In vitro	LPS-induced inflammation in BV-2 cells	1, 5, 10, or 20 µM	Decrease in NO, IL-6, TNF-α, IL-1β, and iNOS	Wang et al. 2015)
Madecassoside	In vivo	MPTP-induced PD in rats	15, 30, or 60 mg/kg	Increase in DA and BDNF. Decrease in MDA. Regulation of Bcl-2 and Bax	Xu et al. 2013)
Triptolide	In vitro	α-Syn-induced neurotoxicity in DA neuron cells	5 or 50 nM	Decrease in aggregation of α-Syn by autophagy. Increase in LC3-II expression	Hu et al. 2017)
**Alkaloids**					
Berberine	In vitro	6-OHDA-induced neurotoxicity in PC12 cells	2, 4, or 8 µM	Increase in cell viability	Wang et al. 2021a)
	In vivo	MPTP-induced neurotoxicity in Zebrafish	50 mg/kg	Improvement in the locomotor activity, Attenuation of NLRP3-mediated neuroinflammation, Reduction in the expression of Casp-1 and IL-1β	Huang et al. 2021)
Caffeine	In vivo	LPS-induced inflammation in mice	30 mg/kg	Decrease in ROS and LPO, Increase in the expression of Nrf2 and HO-1, Decrease in the expression of TLR4, NF-kB, and JNK, Regulation of Bcl2/Bax, Decrease in Casp-3	Badshah et al. 2019)
		α-Syn-induced neurotoxicity in mice	1 g/L in drinking water	Decrease in α-Syn-rich aggregates, cell apoptosis, and activation of microglia and astroglia	Luan et al. 2018)
Piperine	In vivo	6-OHDA-induced PD in mice	Combination of quercetin (20, 40, or 80 mg/kg) and piperine (20 mg/kg)	Enhancement of MAO-B inhibition, increase of DA level, decrease of TNF-α and oxidative stress markers	Rinwa and Kumar 2017)
		MPTP-induced PD in mice	10 mg/kg	Reduction in Casp-3 and -9 activity, Decrease in IL-1β and TNF-α, Regulation of Bcl-2/Bax, Reduction in activated microglia, Increase in TH^+^ neurons	Yang et al. 2015)
		MPTP-induced PD in rats	10 mg/kg	Improvement in the locomotor activity, Reduction in Casp-3 and -9 activity, Decrease in IL-1β and TNF-α, Regulation of Bcl-2/Bax	Shrivastava et al. 2013)
**Flavonoids**					
Apigenin	In vivo	Rotenone-induced PD in rats	20 mg/kg	Suppression of α-Syn aggregation, Decrease in TNF-α and IL-6, Increase in TH expression	Anusha et al. 2017)
Luteolin	In vitro	6-OHDA-induced neurotoxicity in PC12 cells	20 µM	Decrease in ROS, cytotoxicity, and Casp-3, Down-regulation of p53, Up-regulation of HO-1, GCLC, and Nrf2-ARE pathways	Hu et al. 2014)
Baicalein	In vitro	MPTP-induced neurotoxicity in SH-SY5Y cells	50 µM	Increase in cell viability, Decrease in MDA and ROS, Increase in GSH	Song et al. 2021)
		MPTP-induced neurotoxicity in mixed microglial cells	10, 20, or 40 µM	Inhibition of NLRP3 and Casp-1	Rui et al. 2020)
	In vivo	MPTP-induced PD in mice	10 or 50 mg/kg	Improvement in the locomotor activity, Decrease in LPO, Increase in, Decrease in MAO-B	Song et al. 2021)
		MPTP-induced PD in mice	140, 280, or 560 mg/kg	Improvement in motor dysfunction, Decrease in pro-inflammatory cytokines and loss of DA neurons	Rui et al. 2020)
		MPTP-induced PD in mice	1 or 10 mg/kg	Down-regulation of NF-κB, ERK, and JNK	Lee et al. 2014)
Chrysin	In vivo	6-OHDA-induced PD in rats	10 mg/kg	Decrease in TNF-α, IL-1β, and NF-κB 3, Increase in DA and TH	Fabbro et al. 2019)
		MPTP-induced PD in mice	50, 100, or 200 mg/kg	Increase in GSH and SOD, Decrease in LPO, Increase in DA	Krishnamoorthy et al. 2019)
Naringin	In vitro	LPS-induced inflammation in PC12 cells	0–2000 ng/ml	Increase in cell viability, Down-regulation of CYP2E1, Decrease in ROS, Increase in Nrf2, HO-1, SOD, and GSS, Down-regulation of IL-1β, IL-6, TNF-α, COX-2, TLR4, MAPK, and NF-κB, Decrease in Cytc and Casp-3	Wang et al. 2017b)
	In vivo	Rotenone-induced PD in rats	80 mg/kg	Improvement in the locomotor activity, Decrease in cell apoptosis, Maintenance of the functional integrity of mitochondria	Garabadu and Agrawal 2020)
Naringenin	In vitro	6-OHDA-induced neurotoxicity in SH-SY5Y cells	20, 40, or 80 mM	Increase in Nrf2/ARE expression	Lou et al. 2014)
	In vivo	6-OHDA-induced neurotoxicity in mice	70 mg/kg	Decrease in oxidative damage and DA neurons neurodegeneration. Increase in Nrf2/ARE expression	Lou et al. 2014)
Hesperidin	In vitro	Rotenone-induced neurotoxicity in SK-N-SH cells	2.5, 5, 10, 20, or 40 µg	Increase in cell viability and GSH, decrease in ROS and LPO, regulation of MMP, decrease of Cytc release, Casp-3 and -9 activities, decrease in Bax and increase in Bcl-2	Tamilselvam, et al. 2013)
		6-OHDA-induced neurotoxicity in Zebrafish larvae	10, 20, or 40 µM	Improvement in the locomotor activity, down-regulation of lrrk2, gsk3β, polg, and Casp-9	Kesh et al. 2021)
		6-OHDA-induced neurotoxicity in SH-SY5Y cells	12.5 or 25 µM	Increase in cell viability, increase in GSH and SOD, regulation of MMP	Kesh et al. 2021)
	In vivo	6-OHDA-induced PD in mice	50 mg/kg	Reversal of memory impairment. Increase in GPx and catalase activity. Enhancement in the total reactive antioxidant potential and the DA level	Antunes et al. 2014)
		MPTP-induced PD in mice	50 or 100 mg/kg	Improvement in motor potential. Decrease in ROS. Increase in SOD and GPx. Deactivation of microglia. Decrease in COX-2 and inflammatory cytokines	Kabuto and Yamanushi 2011)
Quercetin	In vitro	6-OHDA-induced neurotoxicity in PC12 cells	20 µM	Improvement in mitochondrial potential, decrease in ROS, increase in PINK1 and PRKN, decrease in α-Syn	Wang et al. 2021b)
		6-OHDA-induced neurotoxicity in MN9D cells	10 or 30 µM	Activation of the PKD1-Akt pathway, enhancement of mitochondrial biogenesis	Ay et al. 2017)
	In vivo	6-OHDA-induced PD in rats	50 mg/kg	Increase in DA. Decrease in protein carbonyl content	Haleagrahara et al. 2013)
		Rotenone-induced PD in rats	25–75 mg/kg	Increase in complex I. Repair of mitochondrial electron transport defects. Increase in GSH, GSSG, and SOD	Karuppagounder et al. 2013)
		6-OHDA-induced PD in rats	10 and 30 mg/kg	Improvement in motor behavior. Decrease in neuronal death, mitochondrial damage, and α-Syn	Wang et al. 2021b)
		MitoPark transgenic mice	25 mg/kg	Reversal of behavioral deficits, DA depletion, and TH loss. Activation of the PKD1-Akt pathway	Ay et al. 2017)
Rutin	In vitro	MPTP-induced neurotoxicity in SH-SY5Y cells	25 µM, 50 µM, or 100 µM	Decrease in Casp-3 and -9, regulation of AKT/AMPK pathways, decrease in cleaved PARP and Cytc	Enogieru et al. 2021)
Kaempferol	In vitro	MPTP-induced neurotoxicity in SH-SY5Y cells	30 µM	Decrease in cell apoptosis, decrease in ROS and LPO, decrease in LDH, promotion of lipid droplet autophagy	Han et al. 2021)
	In vivo	MPTP-induced PD in rats	50 mg/kg	Improvement in the locomotor activity. Increase in DA levels	Han et al. 2021)
		SNCA overexpression-induced neurotoxicity in rats	100 mg/kg	Inhibition of NLRP3 inflammatory vesicle activation	Han et al. 2019a)
Fisetin	In vitro	MPTP-induced neurotoxicity in SH-SY5Y cells	2.5 µM	Increase in DA and GSH, decrease in MDA and α-Syn	Rosado-Ramos et al. 2021)
	In vivo	Rotenone-induced PD in rats	10 or 20 mg/kg	Improvement in motor function. Increase in complex I	Alikatte et al. 2021)
Myricitrin	In vivo	6-OHDA-induced PD in mice	60 mg/kg	Increase in DA, TH, mTORC1. Suppression of TNF-α expression	Kim et al. 2016)
Diadzein	In vitro	LPS-induced inflammation in BV-2 cells	25, 50, 75 µM	Reduction in NO, ROS, IL-6, dephosphorylation of p38/MAPK and NF-κB, suppression of microglial activation	Chinta et al. 2013)
**Phenolics**					
Vanillin	In vivo	LPS-induced inflammation in rats	5, 10, or 20 mg/kg	Improvement in the locomotor activity. Decrease in iNOS, COX-2, IL-1β, and IL-6	Yan et al. 2017)
Gallic acid	In vitro	6-OHDA-induced neurotoxicity in SH-SY5Y cells	0.25–2.5 µg/ml	Improvement in the percentage of live cells. Decrease in ROS, Casp-3, and Keap1. Up-regulation of Nrf2 and BDNF	Chandrasekhar et al. 2018)
	In vivo	6-OHDA-induced PD in rats	50, 100, or 200 mg/kg	Increase in the passive avoidance memory. Increase in the total thiol, and GPx. Decrease in MDA	Mansouri et al. 2013)
Ellagic acid	In vivo	6-OHDA-induced PD in rats	50 mg/kg	Improvement of behavior. Decrease in TNF-α and IL-1β levels	Farbood et al. 2015)
		6-OHDA-induced PD in rats	50 mg/kg	Improvement in motor dysfunction. Decrease in MDA and ROS. Prevention of TH^+^ neurons loss. Regulation of ERβ/Nrf2/HO-1 pathway	Baluchnejadmojarad et al. 2017)
		MPTP-induced PD in rats	10 mg/kg	Increase in GSH. Decrease in COX-2 and iNOS	Ardah et al. 2020)
Epigallocatechin gallate	In vitro	LPS-induced inflammation in BV2 cells	25–200 µM	Decrease in NO, TNF-α, and DPPH	Cheng et al. 2021)
	In vivo	MPTP-induced PD in mice	25 and 50 mg/kg	Improvement in motor dysfunction. Increase in TH. Decrease in TNF-α and IL-6. Modulation of the peripheral immune response	Zhou et al. 2018)
		LPS-induced inflammation in rats	10 mg/kg	Restoration of motor function. Decrease in inflammatory cytokines. Decrease in microglia activation	Cheng et al. 2021)
Ferulic acid	In vitro	MPTP-induced neurotoxicity in SH-SY5Y cells	0.3, 1, 3, 10, 30, or 100 µM	Increase in MMP and Nrf2. Regulation of GSH/GSSG and NAD^+^/NADH	Li et al. 2020b)
	In vivo	6-OHDA-induced PD in rats	100 mg/kg	Up-regulation of PGC1α. Down-regulation of BAX, Cytc, p53, and cleaved PARP	Anis et al. 2020)
		MPTP-induced PD in mice	25 mg/kg	Restoration of motor deficits. Decrease of ROS	Li et al. 2020b)
α-Asarone	In vitro	LPS-induced inflammation in BV-2 cells	10, 50, or 250 µM	Inhibition of NF-κB	Kim et al. 2015)
	In vivo	MPTP-induced PD in mice	10 mg/kg	Improvement in motor deficiency using two different behavioral tests	Kim et al. 2015)
Schisandrin B	In vitro	6-OHDA-induced neurotoxicity in SH-SY5Y cells	100 mM	Cell survival; Regulation Nrf2/miR-34a	Ba et al. 2015)
	In vivo	6-OHDA-induced PD in rats	80 mg/kg	Increase in DA. Activation of the Nrf2 pathway	Ba et al. 2015)
Caffeic acid phenethyl ester	In vitro	6-OHDA-induced neurotoxicity in SH-SY5Y cells	1.25 µM	Increase in cell viability. Decrease in ROS. Increase in Bcl-2 and AKT. Reduction in Bax and Casp-9. Regulation of MMP	Turan et al. 2020)
	In vivo	6-OHDA-induced PD in rats	20 or 80 µmol/5 µL/4 day	Enhancement of motor dysfunction in four different behavioral test. Decrease in the loss of TH^+^ neurons	Soner, et al. 2021)
6-Shogaol	In vitro	MPTP-induced neurotoxicity in mesencephalic cultures	0.001 or 0.01 µmol/L	Decrease in TNF-α and IL-1β	Park et al. 2013)
	In vivo	MPTP-induced PD in mice	10 mg/kg	Decrease in TNF-α and IL-1β. Reduction of NO, iNOS, and COX-2	Huh et al. 2020)
		MPTP-induced PD in mice	10 mg/kg	Increase in TH^+^ neurons. Reduction of NO, iNOS, and COX-2	Park et al. 2013)
Salvianolic acid A	In vitro	Rotenone-induced neurotoxicity in SH-SY5Y cells	0.1, 1, or 10 µM	Increase in cell viability. Decrease in ROS	Wang et al. 2020)
	In vivo	Rotenone-induced PD in rats	15, 30, or 60 mg/kg	Improvement in motor dysfunction. Activation of PI3K/AKT/Nrf2 pathway. Decrease in Keap1. Increase in TH	Wang et al. 2020)
		MPTP-induced PD in mice	15 or 60 mg/kg	Improvement in motor dysfunction. Increase in TH. Decrease in IL-1β and TNF-α	Han et al. 2019b)
Magnolol	In vivo	Occlusion-induced ischemia in rats	10 or 30 mg/kg	Reduction in the infarct volume. Decrease in IL-1β, IL-6, and TNF-α. Suppression of iNOS and p38/MAPK. Up-regulation p-Akt	Chen et al. 2014)
Curcumin	In vitro	Rotenone and salsolinol-induced neurotoxicity in SH-SY5Y cells	1–10 µM	Increase in cell viability and decrease in Casp-3 activity	Qualls et al. 2014)
**Quinones**					
Coenzyme Q10	In vivo	Paraquat-induced PD in mice	200 mg/kg	Improvement in the behavioural activity. Decrease in the protein carbonyl content and the mitochondrial damage in the brain	Attia and Maklad 2018)
		6-OHDA-induced PD in rats	25 and 40 µg/mL	Exhibition of a larger number of DA neurons. High expression of angiogenetic factors. Decrease in neuro-inflammation	Park et al. 2020)
		6-OHDA-induced PD in rats	200 mg/kg	Up-regulation and down-regulation of miR-149-5p and MMP-2,9, respectively. Improvement in motor function and increased TH ^+^ cells	Ghasemloo et al. 2021)
Thymoquinone	In vitro	MPTP-induced neurotoxicity in SH-SY5Y cells	10 mM	Increase in cell viability. Increase in Nrf2/ARE and antioxidant enzymes	Dong et al. 2021)
		MPTP-induced neurotoxicity in SH-SY5Y cells	10 mM	Increase in cell viability. Decrease in α-Syn	Ardah et al. 2019)
		α-Syn-induced synaptic toxicity in hiPSC-derived neurons	100 nM	Protection against synapse damage. Reversal of neurons loss	Alhebshi et al. 2014)
	In vivo	6-OHDA-induced PD in rats	5 or 10 mg/kg	Improvement in behavior. Decrease in MDA level and increase in SOD activity	Sedaghat et al. 2014)
		MPTP-induced PD in rats	10 mg/kg	Increase in SOD. Decrease in COX-2 and iNOS levels	Ardah et al. 2019)
**Coumarins**					
Umbelliferone	In vivo	MPTP-induced neurotoxicity in mice	Umbelliferone (0.75 mg/kg) combined with esculetin (1.125 mg/kg)	Attenuation of neurotoxicity in the SN but not striatum. Decrease in ROS. Increase in GSH. Decrease in Casp-3	Subramaniam and Ellis 2013)
**Cannabinoids**					
Cannabidiol	In vitro	Oxygen–glucose deprivation (OGD)-induced ischemia in rat organotypic hippocampal slices	0.1–10 µM	Decrease in neuronal damage, tissue disorganization, and cell death	Landucci et al. 2021)
	In vivo	6-OHDA-induced PD in mice	100 mg/kg	Reduction in hyperalgesia and allodynia. Reduction in nociceptive threshold. Increase in the endogenous anandamide level. Activation of CB1 and CB2 receptors	do Nascimento et al. 2020)
**Fatty acids**					
Docosahexaenoic acid	In vitro	MPP^+^-induced PD in PC12 cells	50, 100, 200 nM	Down-regulation of TNF-α and IL-6. Inhibition of NF-κB protein expression	Xu et al. 2017)
	In vivo	MPTP-induced PD in adult male Wistar rats	36 mg/kg for 30 days	Reduction in latency time in the catalepsy test	Tanriover et al. 2010)

##### Clinical studies

The focus of this review is primarily on the investigation of individual molecules derived from natural products in PD. Although single molecules were preferred due to their well-defined efficacy compared to complex plant extracts, making it easier to study their mechanisms of action and identify similar compounds for further development, there is relatively limited clinical research on natural compounds for PD. Currently, caffeine and curcumin have entered phase II and phase I clinical trials, respectively, targeting motor and non-motor symptoms (Ghodsi et al. 2022; Postuma et al. 2017). Furthermore, nicotine has garnered significant attention among natural compounds in clinical trials for PD therapy, with three completed studies focusing on its neuroprotective effects and improvements in motor and cognitive symptoms (www.ClinicalTrials.gov, Identifier: NCT03865121, NCT02452125, and NCT00873392). Another randomized, double-blind, placebo-controlled phase I/II trial explored the treatment of motor impairments in PD using nicotine. Participants with idiopathic PD (*n* = 65) were divided into two groups and administered either an oral placebo or oral capsules containing nicotine at rising doses of 1 mg to 6 mg four times daily for 10 weeks. The study revealed that nicotine significantly reduced falls and gait freezing, compared to the placebo group. No significant difference in dyskinesia was observed between the groups (Lieberman et al. 2019).

Docosahexaenoic acid (DHA), a type of omega-3 fatty acid, has been reported to reduce dyskinesia in a randomized, multicenter, triple-blind, placebo-controlled phase I study (www.ClinicalTrials.gov, Identifier: NCT01563913). Thirty-three participants were divided into two groups and received a daily dose of 2 g of DHA or a placebo for 18 months. The study demonstrated elevated DHA levels in both plasma and cerebrospinal fluid without significant side effects, indicating the safety and tolerability of the compound. Furthermore, patients receiving DHA exhibited higher levels of reduced dyskinesia compared to the control group.

Epigallocatechin gallate (EGCG) has been evaluated as a neuroprotective agent in de novo PD patients in a randomized, double-blind, placebo-controlled phase II study. The participants (*n* = 480) received EGCG at doses of 0.4, 0.8, or 1.2 g daily given in two equal oral doses or a placebo. The treatment lasted for 1 year, with the placebo group switching to 1.2 g daily of EGCG after 6 months. The rating method used to assess PD progression showed significant improvement in the treatment groups compared to the placebo group at the 6-month mark. However, after a year, the results were no longer significantly different. While EGCG demonstrated symptomatic relief for PD patients, the authors concluded that it did not appear to have noticeable disease-modifying effects (Chan, et al. 2009).

As well, cannabidiol has entered phase II in two studies for managing the motor symptoms of PD (www.ClinicalTrials.gov, Identifier: NCT02818777 and NCT03582137).

The therapeutic effects of Coenzyme Q10 (CoQ10) in patients with PD were investigated in several eligible clinical studies (Strijks et al. 1997; Shults et al. 2002; Müller et al. 2003; Investigators 2007; Storch et al. 2007; Beal et al. 2014; Jie 2014; Wang et al. 2014; Li et al. 2015; Yoritaka et al. 2015). For instance, Yoritaka et al*.* (2016) conducted a study that demonstrated a significant improvement in motor symptoms of PD patients experiencing the “wearing-off” phenomenon (Yoritaka et al. 2015). Similarly, Li et al. (2015) reported a positive effect on cognitive impairment when CoQ10 and creatine were administered together, as assessed by the Montreal Cognitive Assessment. However, it should be noted that these results are based on a small series of patients (Li et al. 2015). In a case report by Mitsui et al. (2017), the effects of CoQ10 treatment (1200 mg/day) were examined in a patient diagnosed with familial multiple system atrophy (MSA) in an advanced stage. The patient had compound heterozygous nonsense (R387X) and missense (V393A) mutations in the COQ2 gene. The administration of CoQ10 resulted in increased serum and cerebrospinal fluid total CoQ10 concentrations, an increased cerebral metabolic ratio of oxygen, and stability in several clinical scores (Barthel Index, Scale for the Assessment and Rating of Ataxia-SARA, International Cooperative Ataxia Rating Scale-ICARS, and the Unified Multiple System Atrophy Rating Scale-UMSARS) during a 3-year follow-up period (Mitsui et al. 2017).

In the context of progressive supranuclear palsy (PSP), two randomized clinical trials investigated the effects of CoQ10. Stamelou et al. (2008) conducted a 6-week, double-blind, placebo-controlled phase II trial involving 21 clinically probable PSP patients. The patients were administered a liquid nano-dispersion of CoQ10 (doses of 5 mg/kg/day) or a placebo. The results showed a mild improvement in the Frontal Assessment Battery and total scores of the PSP rating scale (PSPRS) in the CoQ10 group. However, there were no significant changes in the Unified Parkinson’s Disease Rating Scale (UPDRS) and the Mini-Mental State Examination (MMSE). Adverse effects were not extensively described, and plasma levels of CoQ10 increased in the treated patients. The ratio of high-energy phosphates to low-energy phosphates in specific brain regions also showed significant improvement in the CoQ10 group, suggesting a potential disease-modifying neuroprotective effect (Stamelou et al. 2008). In contrast, Apetauerova et al. (2016) conducted a 1-year, double-blind, placebo-controlled clinical trial involving 61 PSP patients. The participants were assigned to receive CoQ10 (2400 mg/day) or a placebo. The study did not find significant differences between the two groups in PSPRS, although there was a non-significant trend toward a slower decline in the CoQ10 group. The study also assessed UPDRS, activities of daily living (ADL), MMSE, the 39-item Parkinson’s Disease Questionnaire (PDQ-39), and the 36-item Short-Form Health Survey (SF-36), and no significant differences were observed. Although CoQ10 was well-tolerated, a significant number of participants (41%) withdrew from the study for various reasons (Apetauerova, et al. 2016).

##### Structure–activity relationship

Flavonoids exhibit diverse biological activities, and their chemical structure plays a crucial role in determining their effects (Fig. 2). The addition of hydroxyl (-OH) groups to flavonoids has been found to enhance their antioxidant activity. Moreover, longer chain substitutions in flavonoids have been associated with increased activity in 5-HT and NE pathways. Interestingly, the presence of the -OH group at the C3′ position has been shown to reduce the activity of AChE. Additionally, monosubstitution at the C3′ and C4′ positions enhances the inhibition of MAO-A, whereas disubstitution increases the inhibition of MAO-B. Furthermore, the presence of a carbonyl (= O) group is essential for BDNF activity. Conversely, substituting the C3 position with a longer chain has been reported to decrease BDNF activity and the potential for inhibiting MAO enzymes (Pannu et al. 2021). As well, flavonoids with three vicinal hydroxyl groups exhibit the most potent inhibitory effects on α-Syn fibrillation. For instance, myricetin demonstrates stronger inhibition compared to quercetin, and tricetin is more effective than luteolin (Oliveri 2019).

Similar to flavonoids, the total number of hydroxyl groups plays a critical role in determining the α-Syn inhibitory capacity of phenolic acids and catechols **(**Fig. 2**)** (Oliveri 2019). The potency follows the trend: trihydroxybenzoic acid > dihydroxybenzoic acid > monohydroxybenzoic acid. Notably, the presence of three vicinal hydroxyl groups, as in gallic acid, significantly inhibits α-Syn fibrillation (Oliveri 2019). On the other hand, hydrophobicity plays a crucial role in determining the antioxidant activity in cell systems. Hydrophobic antioxidants can easily enter the cytoplasm and attenuate ROS formation and accumulation in 6-OHDA toxicity. For instance, gallic acid exhibits weaker protective effects compared to its esters (Lu et al. 2006).

In context of ginsenosides, spectroscopic techniques have shown that Rb1 does not directly interact with monomeric α-Syn but reasonably stabilizes the structure of soluble oligomeric α-Syn without beta-sheet content (Ardah et al. 2015). SAR studies have indicated that sugar moieties play a significant role in the anti-aggregate capacity of these saponins. Rb1, the most potent inhibitor among the tested ginsenosides, contains four sugar rings, while Rg3, which only partially inhibits α-Syn fibrillation, has two sugar moieties. The authors also hypothesize that the structural symmetry of Rb1, with disaccharide units on each side of the gonane nucleus, contributes to its stronger anti-aggregate properties compared to Rg3, which has only a single disaccharide group attached to C-3 of the triterpene (Oliveri et al. 2015).

On the other side, other terpenoids such as carotenoids, retinoids, and tocopherols exhibit a high degree of hydrophobicity that may be attributed to its interaction with the highly hydrophobic N-terminal acetylation domain of α-Syn (Oliveri 2019).

As for alkaloids **(**Fig. 2**)**, nicotine is a more effective inhibitor for α-Syn fibrillation than structurally similar compounds such as nornicotine, anabasine, and cotinine; the predominant metabolites of nicotine. Recent techniques have established that nicotine binds stereospecifically with a one-site interaction mechanism. Specifically, ( −)-nicotine mediates the interaction between the N- and C-termini of α-Syn, while ( +)-nicotine binds to the N-terminal region with a lower affinity (Tavassoly et al. 2014). On the other hand, it has been reported that caffeine (another alkaloid compound that interacts with α-Syn) and ( −)-nicotine can simultaneously bind to α-Syn, indicating different binding sites for these compounds, showing a distinct and separate mechanism from nicotine (Tavassoly et al. 2014).

#### Volatile compounds

Essential oils (EOs) and their bioactive compounds, known for their aromatic properties, are also being explored for their potential benefits in PD management, particularly in relation to neuroprotection and symptom relief (Abd Rashed et al. 2021). For instance, 1,8-cineole, a saturated monoterpene, is identified as a major component found in various types of EOs, primarily from *Eucalyptus globulus.* (Sadlon and Lamson 2010). Studies have shown that 1,8-cineole possesses potent antioxidant properties and can scavenge free radicals (Euch et al. 2019). As well, one study focused on EO extracted from *Aloysia citrodora Palau* leaves, which showed neuroprotective activity, with a high presence of 1,8-cineole (Abuhamdah et al. 2015).

In addition, a study conducted by Ramazani et al. (2020) evaluated the neuroprotective effects of EO extracted from *Cinnamomum* sp. and cinnamaldehyde, using a PD model induced by 6-OHDA in PC12 cells. Overall, the results determined that the combined actions of cinnamaldehyde and the essential oil may enhance its function for the treatment of PD (Ramazani et al. 2020). As well, an in vitro study focused on *Cuminum cyminum* EO highlighted the inhibitory role of cuminaldehyde in α-Syn fibrillation (Morshedi et al. 2015). Interestingly, cytotoxicity assays indicated no toxic effects with cuminaldehyde treatment during α-Syn fibrillation on PC12 cells (Morshedi et al. 2015).

In addition to in vitro studies, two in vivo studies examined the regulatory effect of β-asarone, isolated from *Acorus* sp., on 6-OHDA-induced PD in rats. The studies focused on the regulation of endoplasmic reticulum (ER) stress pathways, which play a role in protein folding associated with PD (Ning et al. 2016; Ning et al. 2019).

Another study investigated the neuroprotective effects of zingerone (extracted from ginger) and eugenol (derived from cloves) on DA concentration, behavioral changes, and antioxidant activities using 6-OHDA-induced PD animal models (Kabuto et al. 2005; Kabuto et al. 2007; Kabuto and Yamanushi 2011).

In a study by Issa et al. (2020), the neuroprotective effect of *Pulicaria undulata* EO with carvotanacetone as the major component (80.14%), was evaluated in male Wistar rats using a rotenone-induced PD model. The study demonstrated that *P. undulata* EO exerted neuroprotective effects through its anti-inflammatory and antioxidant properties by down-regulating iNOS expression and reducing the gene expression of α-Syn (Issa et al. 2020).

As well, farnesol enhanced the farnesylation process of the protein PARIS, which hindered its ability to repress PGC-1α by reducing the presence of PARIS at the PPARGC1A promoter site. Through the farnesylation of PARIS, farnesol was able to avert the loss of DA-producing neurons as well as behavioral issues in various models, including PARIS transgenic mice, ventral midbrain injected with AAV-PARIS, and adult mice with conditional deletion of the PRKN gene (Jo, et al. 2021).

**Fig. 3:**
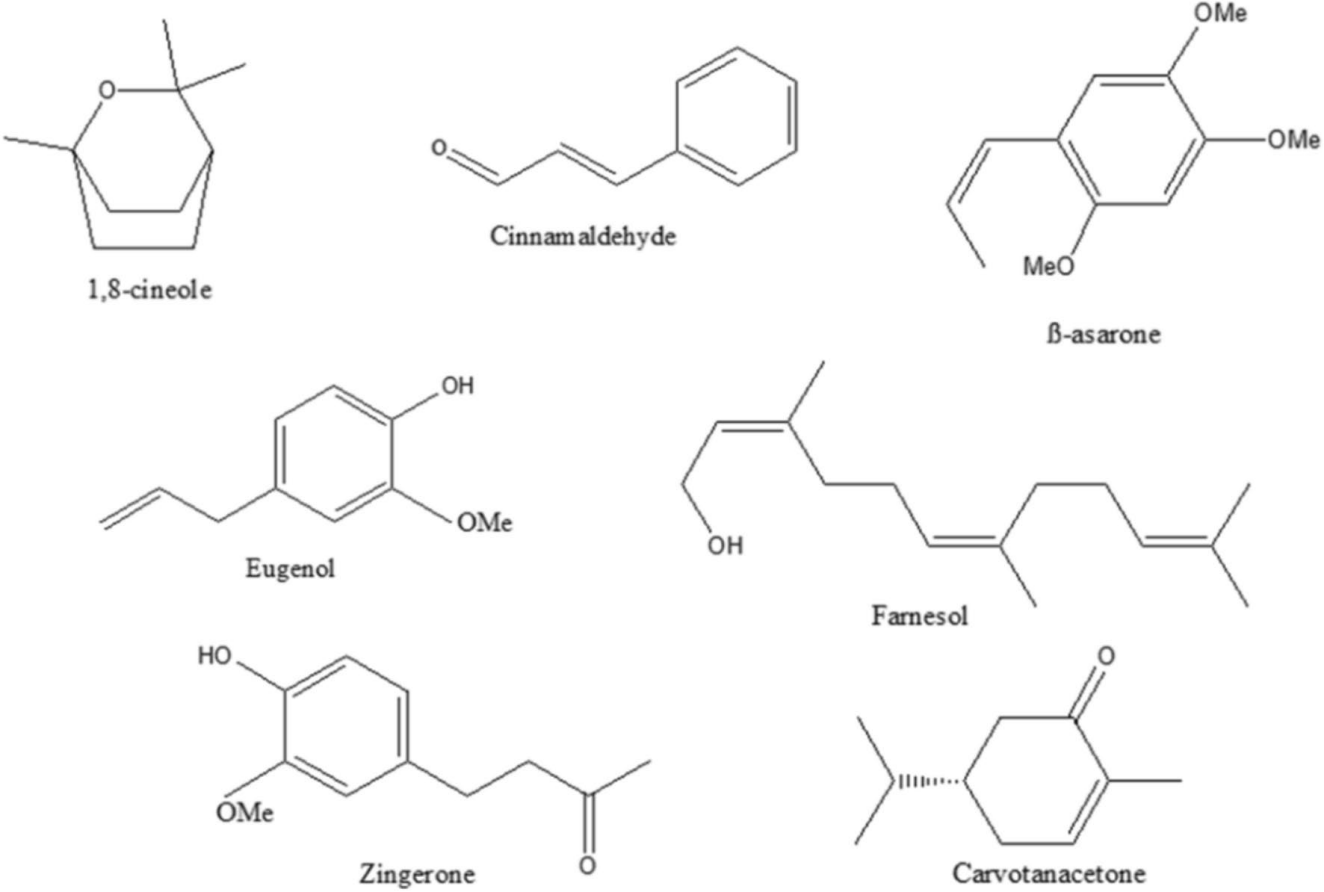
Chemical structures of bioactive compounds from different essential oils that have been reviewed for their anti-PD effects

Chemical structures of the previously discussed volatile compounds are shown in Fig. 3.

## Discussion

In research related to PD, the investigation of natural compounds, animal models, and clinical studies plays a crucial role in understanding the potential therapeutic effects of these compounds on PD. Animal models play a crucial role in experimental medical research, aiding in the enhanced comprehension of the pathogenesis of human diseases (Rai and Singh 2020). Once established, these models can be utilized to assess therapeutic strategies aimed at addressing the functional disruptions witnessed in the specific disease (Betarbet et al. 2002). Drawing from both experimental and clinical data, PD emerged as the pioneering neurological disorder to be replicated in animal models and subsequently treated through neurotransmitter replacement therapy (Jankovic and Tan 2020). Disrupting or damaging catecholaminergic systems, such as those achieved through substances like reserpine, methamphetamine, 6-OHDA, and MPTP, has been instrumental in creating models of PD (Stanford and Heal 2019). More recently, it has been observed that certain agricultural chemicals like rotenone and paraquat, when administered systemically, can replicate specific PD features in rodents, likely through oxidative harm (Lal and Chopra 2024). Transgenic animals engineered to overexpress α-Syn are utilized to explore the role of this protein in the degeneration of dopaminergic cells (Prymaczok, et al. 2024). The central question unifying these models in the pursuit of improved PD drug therapies is the extent to which they mirror the human condition and how reliably they forecast the successful translation of drugs into clinical settings. An ideal PD model would exhibit a high level of construct validity, indicating a similar disease pathogenesis (e.g., involving oxidative stress, inflammation, complex I inhibition, or proteasome inhibition), face validity, reflecting comparable symptoms (e.g., dyskinesia, rigidity), biochemistry (e.g., decreased striatal DA and altered downstream neurochemistry), and pathology (nigrostriatal tract degeneration and Lewy body formation) to the human condition, as well as predictive validity, signifying the capacity to accurately identify clinically effective treatments (Betarbet et al. 2002). For instance, in the late 1950s, Carlsson et al. (1957) initially illustrated the capacity of L-dopa, the natural precursor to DA, to reverse the described tranquilizing effects of reserpine pretreatment in mice (Carlsson et al. 1957). This discovery was swiftly confirmed in humans, establishing reserpine-treated rodents, as a reliable method to evaluate the potential symptomatic benefits of new drugs for PD (Degkwitz et al. 1960). As well, MPTP, a widely used toxin, is effective in inducing Parkinsonism in both rodents and primates, based on its ability to induce persistent symptoms akin to PD in humans (Davis et al. 1979; Langston et al. 1983). Subsequent research in non-human primates revealed that MPTP causes a selective destruction of dopaminergic neurons in the nigrostriatal tract underlying the motor impairments observed, leading to the development of the most relevant animal model of PD that continues to be used today (Burns et al. 1983). In addition, it mirrors the pattern of cell death that seen in humans, with a greater impact on the SN pars compacta compared to the ventral tegmental area (German et al. 1989). Another widely used toxin is 6-OHDA that requires direct injection into the brain as it does not efficiently cross the blood–brain barrier. Upon injection, 6-OHDA is absorbed by dopaminergic neurons through the dopamine transporter (DAT) (Ungerstedt 1968). Current research suggests that once inside dopaminergic neurons, 6-OHDA triggers degeneration through a combination of oxidative stress and mitochondrial respiratory dysfunction. Notably, 6-OHDA readily oxidizes to produce reactive oxygen species, reduces levels of antioxidant enzymes in the striatum, elevates iron levels in the SN, and interacts directly with mitochondrial respiratory chain complexes, resulting in respiratory inhibition and increased oxidative stress (Bagwell and Larsen 2024).

While the experimental models employed in PD have shown connections to human health, it remains a daunting task to translate mechanisms of action observed in in vitro and in vivo studies of natural compounds to clinical outcomes in human trials, where it is a critical step in drug development. Discrepancies between preclinical and clinical findings can arise due to various factors. Issues such as limited bioavailability, metabolic differences, and challenges in dose optimization can impact the efficacy of natural compounds in clinical settings compared to preclinical studies (Oyanna and Clarke 2024). The complexity of human diseases like PD, involving multifactorial interactions, also contributes to discrepancies. Variability in study design, patient characteristics, and outcome measures between preclinical and clinical studies further complicates the translation (Hankenson et al. 2024). Furthermore, Pharmacogenomics plays a significant role in explaining 60% to 90% of the diversity in how antiparkinsonian drugs are processed and their effects on the body (Džoljić et al. 2015). Specifically concerning L-dopa, certain genes like ANKK1, BDNF, LRRK2, and PARK2 are considered pathogenic genes that may impact its effects (Guin et al. 2017). On the other hand, genes such as CCK, CCKAR, CCKBR, DRD1, DRD2, DRD3, DRD4, DRD5, GRIN2A, GRIN2B, HCRT, HOMER1, LMO3, and OPRM1 are known as mechanistic genes whose products can influence the efficacy and safety of L-dopa (Cacabelos et al. 2019). The metabolism of L-dopa involves enzymes encoded by genes like COMT, CYP1A2, CYP2B6, CYP2C19, CYP2D6, CYP3A4, CYP3A5, DBH, DDC, G6PD, MAOB, TH, UGT1A1, and UGT1A9. SLC6A3 plays a crucial role as the primary transporter of L-dopa, while genes like ACE, ACHE, and APOE have pleiotropic effects on the efficacy and safety of L-dopa (Cacabelos et al. 2019). Variants in ADORA2A SNPs and HOMER1 have been linked to L-dopa -induced dyskinesia and psychotic symptoms (Rieck et al. 2015). SLC6A3 has been identified as a genetic factor influencing the response to L-dopa treatment in PD (Moreau et al. 2015). Concerning natural compounds, several genetic investigations have implicated adenosine receptors, particularly adenosine A2A receptor polymorphisms, in individual responses to caffeine concerning neurobehavioral functions (Rétey et al. 2007). The C > T polymorphism in the ADORA2A gene (rs5751876) is a common genetic variation associated with caffeine sensitivity, susceptibility to caffeine-induced insomnia, and anxiety (Byrne et al. 2012).

On the other hand, natural compounds combined with conventional therapies for PD, may demonstrate evidence of antagonistic, synergistic, or additive effects. For instance, St. John’s Wort, used for depression, may interact with medications like L-dopa, reducing their effectiveness (Williamson 2003). In a PD model, the combination of palmitoylethanolamide with luteolin has been shown to reduce neuroinflammation and promote autophagy (Cordaro et al. 2020). Interestingly, fava beans (*Mucuna pruriens*), traditionally used in Ayurvedic medicine as it reduces iNOS expression in Parkinsonian mice model (Yadav et al. 2017), contain L-dopa, isolated from their seeds (Rijntjes 2019). Studies have confirmed measurable plasma L-dopa levels post-fava bean ingestion, suggesting clinical activity (Rabey et al. 1993). As well, an animal model of PD revealed *M. pruriens* antioxidant properties, including the scavenging of reactive oxygen species and iron-chelating activity, hinting at a potential neuroprotective role. However, the advantage of using this natural product over commercial L-dopa preparations remains unclear (Tharakan et al. 2007). Additionally, in a separate study on rats, the comparative benefits of a water extract of *M. pruriens* seed powder extract (MPE) were assessed against L-dopa. Both MPE and L-dopa improved parkinsonism but led to dose-dependent drug-induced dyskinesia (DID) when combined with dopa-decarboxylase inhibitor (DDCI) and benserazide (BZ). At a lower dose, MPE + BZ significantly reduced parkinsonism without inducing DID, suggesting a potential advantage over L-dopa. Moreover, MPE surpassed an equivalent dose of synthetic L-dopa alone, offering prolonged anti-PD benefits without DID induction when administered alone without additives. Notably, in animals primed with L-dopa + BZ, MPE alone mitigated DID severity. These findings imply that MPE may contain water-soluble components with inherent dopa-decarboxylase inhibitory-like activity or the ability to reduce the need for an additional DDCI (Lieu et al. 2010). In addition, Hemiparkinsonian monkeys treated with MPE, L-dopa with carbidopa (CD), and MPE with CD were assessed for their effects on parkinsonism. Both MPE and L-dopa with CD effectively treated parkinsonism, suggesting MPE’s potential as an alternative therapy. Neurophysiological assessments of the SN reticulata (SNR) and subthalamic nucleus (STN) revealed differences between MPE and L-dopa treatments. L-dopa with CD enhanced SNR bursting firing patterns, a trait not observed with MPE and CD treatments (Lieu et al. 2012).

### Challenges and future directions

This detailed review article focusing on natural compounds and their neuroprotective role in PD can provide valuable insights into their potential management options and mechanisms of action. Although, certain studies indicate the superior effectiveness of BHP (botanical health products) when compared to single drugs, some pre-planned combinations may prove ineffective, likely due to antagonistic interactions and communication between molecular targets within the intricate networks involved in cellular responses and the overall reactions of organisms to interventions (Panossian et al. 2024). Reviewing the literature for single natural compounds, rather than natural plant extracts, can offer a more precise understanding of their specific effects and target pathways. Recently, network pharmacology studies aid in forecasting study outcomes for discovering new applications and unforeseen adverse effects (Hopkins 2008).

Recent research has indeed highlighted the potential of screening plant extracts for their metabolome using liquid chromatography-mass spectrometry (LC–MS) techniques. LC–MS analysis enables the identification and characterization of individual compounds present in the extract, providing valuable information about their chemical composition. This information allows for the targeted application of the extract or isolated compounds to treat specific diseases. In PD research, natural compounds identified through LC–MS techniques from *Yucca aloifolia* extract unveiled the presence of anthocyanins, saponins, and phenolics. Biochemical and histopathological assessments revealed dose-dependent improvements with oral *Y. aloifolia* extract, suggesting a potential neuroprotective effect for PD (Ali et al. 2023). Furthermore, investigations into *Eucommia ulmoides* leaves extract indicated therapeutic benefits for PD, with HPLC-Q-TOF–MS identifying 28 compounds, including phenolic acids, flavonoids, and iridoids. *E. ulmoides* extract demonstrated significant reversals in dopaminergic neuron loss and neural vasculature, reducing apoptotic cells in a zebrafish PD model, potentially through autophagy activation and α-syn degradation (Zhang et al. 2020).

Bridging the gap between laboratory studies (in vitro) and animal studies (in vivo) with clinical trials is essential for the successful translation of findings. It can be challenging to extrapolate results from cell or animal models to human patients due to inherent differences in biological systems. For this case, there is a need for a deeper mechanistic understanding of how these compounds interact with the complex molecular pathways involved in PD progression. On the other hand, continuous research on recent targets and biomarkers for PD is of utmost importance. For instance, the adenosine A2A receptor, known to modulate neurotransmitter release and neuroinflammation, has emerged as a potential target for neuroprotection in PD (Prasad et al. 2024). Activation of the PGC-1α pathway, involved in mitochondrial biogenesis and oxidative stress regulation, has shown neuroprotective effects in PD models (Mesarosova et al. 2024). The IL-33 cytokine, implicated in immune response regulation and neuroinflammation, exhibits potential neuroprotective target for PD (Aguiar et al. 2016). The DJ-1 gene, associated with oxidative stress response and mitochondrial function, is another promising target for neuroprotection in PD (Skou et al. 2024). Furthermore, the MALAT1/miR-129/SNCA pathway, involved in α-Syn regulation and neuroinflammation, has shown relevance in PD pathogenesis (Thangavelu, et al. 2024).

As well, recent methodologies in PD research have shifted from focusing solely on dopamine-replenishing symptomatic therapies to personalized therapeutics aimed at restoring the molecular, anatomical, and functional integrity of specific brain circuits affected by the disease. This shift has been facilitated by significant technological and methodological advancements that hold great promise for advancing PD research and bridging the existing gap in disease-modifying therapeutics that are personalized to the needs of each patient. For instance, advanced imaging techniques (like functional MRI and PET scans), genetic sequencing, wearable sensors, and big data analytics have played a pivotal role in enabling the transition towards personalized therapeutics in PD. These innovations allow for the precise characterization of individual disease profiles, including motor and non-motor symptoms, genetic predispositions, and neuroimaging markers, facilitating tailored treatment strategies (Iqbal, et al. 2024). For instance, by combining genetic information with neuroimaging data, clinicians can identify dysfunctional brain circuits and tailor interventions, such as deep brain stimulation or targeted drug therapies, to restore the integrity of these circuits (Lu 2024). This personalized approach not only improves treatment outcomes but also enhances the overall quality of life for individuals with PD by addressing their unique needs and optimizing therapeutic efficacy.

Although preclinical studies have shown promise, there is a lack of comprehensive clinical data on the efficacy, safety, and optimal dosages of natural compounds in PD patients. Future clinical trials should address these gaps to assess their effectiveness and potential interactions with standard PD medications. When considering the long-term use of natural compounds in the management of PD, potential side effects and safety concerns must be carefully evaluated. For instance, curcumin, a common natural compound known for its anti-inflammatory properties, can trigger gastrointestinal issues like nausea and diarrhea (Hewlings and Kalman 2017). Long-term consumption of green tea extract, prized for its antioxidants, has been linked to liver toxicity in some cases (Orhan et al. 2021), while *Ginkgo biloba*, popular for cognitive enhancement, has been associated with an increased risk of seizures (Wilson and Maulik 2018). Ginseng, often used for energy, can impact blood pressure, particularly in individuals with specific health conditions (Cui et al. 2006). Moreover, the lack of standardization in herbal supplements can lead to variability in active ingredient concentration, posing inconsistencies and possible safety risks.

Additionally, research focusing on improving the bioavailability and formulation of these compounds is essential to ensure therapeutic concentrations reach the brain. Formulating natural compounds into delivery systems that enhance their bioavailability, protect them from degradation, and promote targeted delivery to the brain is crucial. Recent studies have addressed these challenges through the development of nanoformulations, liposomal delivery systems, and prodrug strategies to improve the pharmacokinetic properties and stability of natural compounds for PD treatment. These innovative approaches aim to overcome the limitations of natural compound-based therapies and optimize their therapeutic benefits for neuroprotection in PD patients.

These challenges and future directions highlight the importance of collaboration between scientists and clinicians in the context of interdisciplinary research and multidisciplinary research. Multidisciplinary and interdisciplinary research both involve collaboration across multiple academic disciplines, but they differ in their integration levels. Multidisciplinary research features researchers working independently on a common problem, contributing their unique insights without deep integration, resulting in separate findings. In contrast, interdisciplinary research emphasizes active collaboration and integration of knowledge, methods, and theories from various fields to create new insights and solutions (Aditya Rao and Shetty 2024). By integrating diverse perspectives and methodologies, researchers have been able to explore the complex mechanisms underlying neurodegeneration in PD and identify potential therapeutic targets for natural compounds. For instance, computational modeling and bioinformatics analyses have been used to predict the interactions between natural compounds and specific molecular targets involved in PD pathogenesis, guiding the design of targeted interventions (Akki et al. 2024). Additionally, advancements in drug delivery systems have significantly enhanced the targeting and pharmacokinetics of natural compounds for PD, offering improved efficacy and reduced side effects. Nanoformulations, liposomal delivery systems, and prodrug strategies have been developed to overcome the bioavailability and stability challenges associated with natural compounds (Yergök et al. 2024). These systems can encapsulate natural compounds, protect them from degradation, and facilitate their targeted delivery to the brain, enhancing their therapeutic effects in PD. For instance, curcumin, resveratrol, ginsenosides, quercetin, and catechin are plant-derived bioactive substances known for their significant roles in preventing and treating PD. Nonetheless, studies conducted in living organisms indicate that their concentrations are often insufficient to effectively traverse the blood–brain barrier, limiting their bioavailability, stability, and dissolution at the intended brain sites. To address these challenges, nanophytomedicine, featuring sizes between 1 and 100 nm, is employed to enhance the efficiency of PD treatment. By reducing the size of these plant-derived bioactive compounds to the nanoscale, their ability to permeate the brain is amplified, leading to increased effectiveness and stability. For instance, nanocompounds like ginsenosides, synthesized at 19.9 nm using a nanoemulsion approach, have demonstrated enhanced bioavailability in the brains of rats (Ganesan 2015). A range of studies have shown that nanoformulations and microneedles containing natural compounds such as quercetin, curcumin, resveratrol, chrysin, piperine, ferulic acid, huperzine A, berberine, baicalein, hesperetin, and retinoic acid have effectively ameliorated various neurodegenerative disorders (Aspatwar et al. 2024).

## Conclusion

This review distinctively highlighted the in vitro, in vivo, and clinical studies conducted on the neuroprotective potential of natural compounds in Parkinson’s disease (PD), discussing the various models of PD used, specified concentrations or doses of natural compounds, and the mechanism of their actions. The evidence suggests that certain natural compounds exhibit promising effects in attenuating neurodegeneration, modulating key cellular pathways, and improving motor function in preclinical models. However, further research, including well-designed clinical trials, is necessary to determine the neuroprotective efficacy and safety of natural compounds in PD. As well, SAR studies help in identifying the critical structural elements necessary for compounds to exhibit desired pharmacological effects. This information enables researchers to optimize the chemical structure of existing compounds or design new molecules with enhanced potency, selectivity, and safety profiles. The integration of multidisciplinary approaches, collaboration among researchers, and a comprehensive understanding of the underlying mechanisms will contribute to the development of novel natural compound-based interventions for PD.

## Data Availability

The manuscript contains all the necessary data and material.

## References

[CR1] Aarsland D et al (2021) Parkinson disease-associated cognitive impairment. Nat Rev Dis Primers 7(1):47 34210995 10.1038/s41572-021-00280-3

[CR2] Abrishamdar M, Jalali M, Rashno M (2022) MALAT1 lncRNA and Parkinson’s disease: the role in the pathophysiology and significance for diagnostic and therapeutic approaches. Mol Neurobiol 59(9):5253–5262 35665903 10.1007/s12035-022-02899-z

[CR3] Abubakar I, Tillmann T, Banerjee A (2015) Global, regional, and national age-sex specific all-cause and cause-specific mortality for 240 causes of death, 1990–2013: a systematic analysis for the Global Burden of Disease Study 2013. Lancet 385(9963):117–171 25530442 10.1016/S0140-6736(14)61682-2 PMC4340604

[CR4] Abuhamdah S et al (2015) Pharmacological and neuroprotective profile of an essential oil derived from leaves of A loysia citrodora Palau. J Pharm Pharmacol 67(9):1306–1315 25877296 10.1111/jphp.12424

[CR5] Aditya Rao SJ, Shetty NP (2024) Advances in designing next-generation drugs from natural products. Authorea Preprints

[CR6] Aguiar AS et al (2016) Moderate-intensity physical exercise protects against experimental 6-hydroxydopamine-induced hemiparkinsonism through Nrf2-antioxidant response element pathway. Neurochem Res 41:64–72 26323504 10.1007/s11064-015-1709-8

[CR7] Akki AJ et al (2024) Advances in Parkinson’s disease research–a computational network pharmacological approach. Int Immunopharmacol 139:112758 39067399 10.1016/j.intimp.2024.112758

[CR8] Alhebshi A et al (2014) Thymoquinone protects cultured hippocampal and human induced pluripotent stem cells-derived neurons against α-synuclein-induced synapse damage. Neurosci Lett 570:126–131 24080376 10.1016/j.neulet.2013.09.049

[CR9] Ali DE et al (2023) HR LC-MS/MS metabolomic profiling of Yucca aloifolia fruit and the potential neuroprotective effect on rotenone-induced Parkinson’s disease in rats. PLoS ONE 18(2):e0282246 36854038 10.1371/journal.pone.0282246 PMC9974117

[CR10] Ali D et al (2024) Implications of herbal components in the treatment of neurological disorders. Curr Nutr Food Sci 20(6):677–686

[CR11] Alikatte K et al (2021) Fisetin improved rotenone-induced behavioral deficits, oxidative changes, and mitochondrial dysfunctions in rat model of Parkinson’s disease. J Diet Suppl 18(1):57–71 31992104 10.1080/19390211.2019.1710646

[CR12] Alosaimi F et al (2022) The role of neurotransmitter systems in mediating deep brain stimulation effects in Parkinson’s disease. Front Neurosci 16:998932 36278000 10.3389/fnins.2022.998932 PMC9579467

[CR13] Anis E et al (2020) Ferulic acid reinstates mitochondrial dynamics through PGC1α expression modulation in 6-hydroxydopamine lesioned rats. Phytother Res 34(1):214–226 31657074 10.1002/ptr.6523

[CR14] Antunes MS et al (2014) Protective effect of hesperidin in a model of Parkinson’s disease induced by 6-hydroxydopamine in aged mice. Nutrition 30(11–12):1415–1422 25280422 10.1016/j.nut.2014.03.024

[CR15] Anusha C, Sumathi T, Joseph LD (2017) Protective role of apigenin on rotenone induced rat model of Parkinson’s disease: suppression of neuroinflammation and oxidative stress mediated apoptosis. Chem Biol Interact 269:67–79 28389404 10.1016/j.cbi.2017.03.016

[CR16] Apetauerova D et al (2016) CoQ10 in progressive supranuclear palsy: a randomized, placebo-controlled, double-blind trial. Neurol: Neuroimmunol Neuroinflam **3**(5):e266 10.1212/NXI.0000000000000266 PMC4990260 27583276

[CR17] Ardah MT et al (2015) Ginsenoside Rb1 inhibits fibrillation and toxicity of alpha-synuclein and disaggregates preformed fibrils. Neurobiol Dis 74:89–101 25449909 10.1016/j.nbd.2014.11.007 PMC4882765

[CR18] Ardah MT, Merghani MM, Haque ME (2019) Thymoquinone prevents neurodegeneration against MPTP in vivo and modulates α-synuclein aggregation in vitro. Neurochem Int 128:115–126 31028778 10.1016/j.neuint.2019.04.014

[CR19] Ardah MT et al (2020) Ellagic acid prevents dopamine neuron degeneration from oxidative stress and neuroinflammation in MPTP model of Parkinson’s disease. Biomolecules 10(11):1519 33172035 10.3390/biom10111519 PMC7694688

[CR20] Aspatwar A et al (2024) Herbal-based nanosystems: a novel drug-delivery treatment procedure against neurodegenerative disorders. ChemRxiv preprints

[CR21] Attia HN, Maklad YA (2018) Neuroprotective effects of coenzyme Q10 on paraquat-induced Parkinson’s disease in experimental animals. Behav Pharmacol 29(1):79–86 28902670 10.1097/FBP.0000000000000342

[CR22] Ay M et al (2017) Molecular mechanisms underlying protective effects of quercetin against mitochondrial dysfunction and progressive dopaminergic neurodegeneration in cell culture and MitoPark transgenic mouse models of Parkinson’s disease. J Neurochem 141(5):766–782 28376279 10.1111/jnc.14033 PMC5643047

[CR23] Ba Q et al (2015) Schisandrin B shows neuroprotective effect in 6-OHDA-induced Parkinson’s disease via inhibiting the negative modulation of miR-34a on Nrf2 pathway. Biomed Pharmacother 75:165–172 26282218 10.1016/j.biopha.2015.07.034

[CR24] Badshah H et al (2019) Caffeine may abrogate LPS-induced oxidative stress and neuroinflammation by regulating Nrf2/TLR4 in adult mouse brains. Biomolecules 9(11):719 31717470 10.3390/biom9110719 PMC6921022

[CR25] Bagwell E, Larsen J (2024) A review of MPTP-induced parkinsonism in adult zebrafish to explore pharmacological interventions for human Parkinson’s disease. Front Neurosci 18:1451845 39170675 10.3389/fnins.2024.1451845 PMC11335677

[CR26] Balakrishnan R et al (2021) Natural phytochemicals as novel therapeutic strategies to prevent and treat Parkinson’s disease: current knowledge and future perspectives. Oxid Med Cell Longev 2021:1–32 10.1155/2021/6680935 PMC8169248 34122727

[CR27] Baluchnejadmojarad T et al (2017) Ellagic acid exerts protective effect in intrastriatal 6-hydroxydopamine rat model of Parkinson’s disease: possible involvement of ERβ/Nrf2/HO-1 signaling. Brain Res 1662:23–30 28238669 10.1016/j.brainres.2017.02.021

[CR28] Bandres-Ciga S et al (2020) Genetics of Parkinson’s disease: an introspection of its journey towards precision medicine. Neurobiol Dis 137:104782 31991247 10.1016/j.nbd.2020.104782 PMC7064061

[CR29] Beal MF et al (2014) A randomized clinical trial of high-dosage coenzyme Q10 in early Parkinson disease: no evidence of benefit. JAMA Neurol 71(5):543–552 24664227 10.1001/jamaneurol.2014.131

[CR30] Behl T et al (2021) Multifaceted role of matrix metalloproteinases in neurodegenerative diseases: pathophysiological and therapeutic perspectives. Int J Mol Sci 22(3):1413 33573368 10.3390/ijms22031413 PMC7866808

[CR31] Bennett CF, Latorre-Muro P, Puigserver P (2022) Mechanisms of mitochondrial respiratory adaptation. Nat Rev Mol Cell Biol 23(12):817–835 35804199 10.1038/s41580-022-00506-6 PMC9926497

[CR32] Betarbet R, Sherer TB, Greenamyre JT (2002) *Animal models of Parkinson*’*s disease*. BioEssays 24(4):308–318 11948617 10.1002/bies.10067

[CR33] Bloem BR, Okun MS, Klein C (2021) Parkinson’s disease. The Lancet 397(10291):2284–2303 10.1016/S0140-6736(21)00218-X 33848468

[CR34] Blum D et al (2001) Molecular pathways involved in the neurotoxicity of 6-OHDA, dopamine and MPTP: contribution to the apoptotic theory in Parkinson’s disease. Prog Neurobiol 65(2):135–172 11403877 10.1016/s0301-0082(01)00003-x

[CR35] Booth S (2024) Limitations in effective treatment of Parkinson’s disease: neuroanatomical substrate of L-Dopa induced dyskinesia and cognitive impairment [Doctoral dissertation, University of Manitoba]. The University of Manitoba's online repository for scholarly works

[CR36] Bové J et al (2005) Toxin-induced models of Parkinson’s disease. NeuroRx 2(3):484–494 16389312 10.1602/neurorx.2.3.484 PMC1144492

[CR37] Burns RS et al (1983) A primate model of parkinsonism: selective destruction of dopaminergic neurons in the pars compacta of the substantia nigra by N-methyl-4-phenyl-1, 2, 3, 6-tetrahydropyridine. Proc Natl Acad Sci 80(14):4546–4550 6192438 10.1073/pnas.80.14.4546 PMC384076

[CR38] Byrne EM et al (2012) A genome-wide association study of caffeine-related sleep disturbance: confirmation of a role for a common variant in the adenosine receptor. Sleep 35(7):967–975 22754043 10.5665/sleep.1962 PMC3369232

[CR39] Cacabelos R, Cacabelos P, Carril JC (2019) Epigenetics and pharmacoepigenetics of age-related neurodegenerative disorders. Pharmacoepigenetics. Elsevier, pp 903–950

[CR40] Carlsson A, Lindqvist M, Magnusson T (1957) 3, 4-Dihydroxyphenylalanine and 5-hydroxytryptophan as reserpine antagonists. Nature 180(4596):1200–1200 13483658 10.1038/1801200a0

[CR41] Chan P et al (2009) P2. 204 A randomized, double-blind, placebo-controlled, delayed start study to assess safty, tolerability and efflcacy of green tea polyphenols in Parkinson’s disease. Parkinsonism Relat Disord 15:S145

[CR42] Chandrasekhar Y et al (2018) Gallic acid protects 6-OHDA induced neurotoxicity by attenuating oxidative stress in human dopaminergic cell line. Neurochem Res 43:1150–1160 29671234 10.1007/s11064-018-2530-y

[CR43] Chao P-C, Lee H-L, Yin M-C (2016) Asiatic acid attenuated apoptotic and inflammatory stress in the striatum of MPTP-treated mice. Food Funct 7(4):1999–2005 26999333 10.1039/c6fo00041j

[CR44] Chen J-H et al (2014) Magnolol protects neurons against ischemia injury via the downregulation of p38/MAPK, CHOP and nitrotyrosine. Toxicol Appl Pharmacol 279(3):294–302 25038313 10.1016/j.taap.2014.07.005

[CR45] Chen D et al (2019) Asiatic acid protects dopaminergic neurons from neuroinflammation by suppressing mitochondrial ROS production. Biomolecules Therapeutics 27(5):442 30971058 10.4062/biomolther.2018.188 PMC6720531

[CR46] Cheng C-Y et al (2021) Epigallocatechin-3-gallate-loaded liposomes favor anti-inflammation of microglia cells and promote neuroprotection. Int J Mol Sci 22(6):3037 33809762 10.3390/ijms22063037 PMC8002297

[CR47] Chinta SJ et al (2013) Anti-inflammatory role of the isoflavone diadzein in lipopolysaccharide-stimulated microglia: implications for Parkinson’s disease. Neurotox Res 23:145–153 22573480 10.1007/s12640-012-9328-5 PMC3597389

[CR48] Colle D, Farina M (2021) Oxidative stress in paraquat-induced damage to nervous tissues. Toxicology. Elsevier, pp 69–78

[CR49] Cordaro M, Cuzzocrea S, Crupi R (2020) An update of palmitoylethanolamide and luteolin effects in preclinical and clinical studies of neuroinflammatory events. Antioxidants 9(3):216 32150935 10.3390/antiox9030216 PMC7139331

[CR50] Cui Y et al (2006) Association of ginseng use with survival and quality of life among breast cancer patients. Am J Epidemiol 163(7):645–653 16484447 10.1093/aje/kwj087

[CR51] Davis GC et al (1979) Chronic Parkinsonism secondary to intravenous injection of meperidine analogues. Psychiatry Res 1(3):249–254 298352 10.1016/0165-1781(79)90006-4

[CR52] Degkwitz R et al (1960) On the effects of L-dopa in man and their modification by reserpine, chlorpromazine, iproniazid and vitamin B6. Klin Wochenschr 38:120–123 13815400 10.1007/BF02189076

[CR53] Del Fabbro L et al (2019) Chrysin protects against behavioral, cognitive and neurochemical alterations in a 6-hydroxydopamine model of Parkinson’s disease. Neurosci Lett 706:158–163 31121284 10.1016/j.neulet.2019.05.036

[CR54] Dettmer U, Selkoe D, Bartels T (2016) New insights into cellular α-synuclein homeostasis in health and disease. Curr Opin Neurobiol 36:15–22 26282834 10.1016/j.conb.2015.07.007

[CR55] do Nascimento GC et al (2020) Cannabidiol increases the nociceptive threshold in a preclinical model of Parkinson’s disease. Neuropharmacology 163:107808 10.1016/j.neuropharm.2019.107808 31706993

[CR56] Doherty KM et al (2013) Parkin disease: a clinicopathologic entity? JAMA Neurol 70(5):571–579 23459986 10.1001/jamaneurol.2013.172 PMC4202385

[CR57] Domingo A, Klein C (2018) Genetics of Parkinson disease. Handbook of clinical neurology. Elsevier, pp 211–227 10.1016/B978-0-444-63233-3.00014-2 29325612

[CR58] Dong J et al (2021) Thymoquinone prevents dopaminergic neurodegeneration by attenuating oxidative stress via the Nrf2/ARE pathway. Front Pharmacol 11:615598 33519481 10.3389/fphar.2020.615598 PMC7840486

[CR59] Dorsey E et al (2018) The emerging evidence of the Parkinson pandemic. J Parkinsons Dis 8(s1):S3–S8 30584159 10.3233/JPD-181474 PMC6311367

[CR60] Džoljić E et al (2015) Pharmacogenetics of drug response in Parkinson’s disease. Int J Neurosci 125(9):635–644 25226559 10.3109/00207454.2014.963851

[CR61] El Euch SK et al (2019) Salvia officinalis essential oil: chemical analysis and evaluation of anti-enzymatic and antioxidant bioactivities. S Afr J Bot 120:253–260

[CR62] Enogieru AB et al (2021) Regulation of AKT/AMPK signaling, autophagy and mitigation of apoptosis in rutin-pretreated SH-SY5Y cells exposed to MPP+. Metab Brain Dis 36:315–326 33146846 10.1007/s11011-020-00641-z

[CR63] Essa M et al (2014) Review of natural products on Parkinson’s disease pathology. J Aging Res Clin Pract 3(1):1–8

[CR64] Farbood Y et al (2015) Ellagic acid protects the brain against 6-hydroxydopamine induced neuroinflammation in a rat model of Parkinson’s disease. Basic Clin Neurosci 6(2):83 27307952 PMC4636882

[CR65] Fields CR, Bengoa-Vergniory N, Wade-Martins R (2019) Targeting alpha-synuclein as a therapy for Parkinson’s disease. Front Mol Neurosci 12:299 31866823 10.3389/fnmol.2019.00299 PMC6906193

[CR66] Franco-Iborra S, Vila M, Perier C (2016) The Parkinson disease mitochondrial hypothesis: where are we at? Neuroscientist 22(3):266–277 25761946 10.1177/1073858415574600

[CR67] Fritz M et al (2016) Prostaglandin-dependent modulation of dopaminergic neurotransmission elicits inflammation-induced aversion in mice. J Clin Investig 126(2):695–705 26690700 10.1172/JCI83844 PMC4731170

[CR68] Ganesan P et al (2015) Recent trends in the development of nanophytobioactive compounds and delivery systems for their possible role in reducing oxidative stress in Parkinson’s disease models. Int J Nanomed 6757–6772. 10.2147/IJN.S93918 10.2147/IJN.S93918 PMC4631432 26604750

[CR69] Garabadu D, Agrawal N (2020) Naringin exhibits neuroprotection against rotenone-induced neurotoxicity in experimental rodents. NeuroMol Med 22(2):314–330 10.1007/s12017-019-08590-2 31916219

[CR70] German DC et al (1989) Midbrain dopaminergic cell loss in Parkinson’s disease: computer visualization. Annals Neurol: Off J Am Neurol Assoc Child Neurol Soc 26(4):507–514 10.1002/ana.410260403 2817827

[CR71] Gershanik OS (2015) Improving l-dopa therapy: The development of enzyme inhibitors. Mov Disord 30(1):103–113 25335824 10.1002/mds.26050

[CR72] Ghasemloo E et al (2021) Neuroprotective effects of coenzyme Q10 in Parkinson’s model via a novel Q10/miR-149-5p/MMPs pathway. Metab Brain Dis 36(7):2089–2100 34357552 10.1007/s11011-021-00795-4

[CR73] Ghodsi H et al (2022) Evaluation of curcumin as add-on therapy in patients with Parkinson’s disease: a pilot randomized, triple-blind, placebo-controlled trial. Clin Neurol Neurosurg 218:107300 35636380 10.1016/j.clineuro.2022.107300

[CR74] Goldenberg MM (2008) Medical management of Parkinson’s disease. Pharm Ther 33(10):590 PMC2730785 19750042

[CR75] Golpich M et al (2015) Glycogen synthase kinase-3 beta (GSK-3β) signaling: implications for Parkinson’s disease. Pharmacol Res 97:16–26 25829335 10.1016/j.phrs.2015.03.010

[CR76] Grünewald A, Kumar KR, Sue CM (2019) New insights into the complex role of mitochondria in Parkinson’s disease. Prog Neurobiol 177:73–93 30219247 10.1016/j.pneurobio.2018.09.003

[CR77] Guin D et al (2017) A systematic review and integrative approach to decode the common molecular link between levodopa response and Parkinson’s disease. BMC Med Genomics 10:1–21 28927418 10.1186/s12920-017-0291-0 PMC5606117

[CR78] Haleagrahara N, Siew CJ, Ponnusamy K (2013) Effect of quercetin and desferrioxamine on 6-hydroxydopamine (6-OHDA) induced neurotoxicity in striatum of rats. J Toxicol Sci 38(1):25–33 23358137 10.2131/jts.38.25

[CR79] Han J-Y, Kim J-S, Son JH (2014) Mitochondrial homeostasis molecules: regulation by a trio of recessive Parkinson’s disease genes. Exp Neurobiol 23(4):345 25548534 10.5607/en.2014.23.4.345 PMC4276805

[CR80] Han X et al (2019a) Small molecule-driven NLRP3 inflammation inhibition via interplay between ubiquitination and autophagy: implications for Parkinson disease. Autophagy 15(11):1860–1881 30966861 10.1080/15548627.2019.1596481 PMC6844502

[CR81] Han B et al (2019b) Neuroprotective effects of Danshensu in Parkinson’s disease mouse model induced by 1-methyl-4-phenyl-1, 2, 3, 6-tetrahydropyridine. Behav Pharmacol 30(1):36–44 29847337 10.1097/FBP.0000000000000412

[CR82] Han X et al (2021) Kaempferol alleviates LD-mitochondrial damage by promoting autophagy: implications in Parkinson’s disease. Redox Biol 41:101911 33713908 10.1016/j.redox.2021.101911 PMC7967038

[CR83] Hankenson F, Prager E, Berridge B (2024) Advocating for generalizability: accepting inherent variability in translation of animal research outcomes. Annual Rev Animal Biosci 12(1):391–410 10.1146/annurev-animal-021022-043531 38358839

[CR84] Hartmann-Nardin D et al (2024) Cost-effectiveness analyses of non-pharmacological and non-surgical interventions in idiopathic Parkinson’s disease: a systematic review. J Parkinson’s Dis (Preprint) 14:1–12 10.3233/JPD-230213 PMC11380296 38339939

[CR85] Hatcher JM, Pennell KD, Miller GW (2008) *Parkinson*’*s disease and pesticides: a toxicological perspective*. Trends Pharmacol Sci 29(6):322–329 18453001 10.1016/j.tips.2008.03.007 PMC5683846

[CR86] He S et al (2024) Advances in animal models of Parkinson’s disease. Brain Res Bulletin 215:111024 10.1016/j.brainresbull.2024.111024 38969066

[CR87] Heinemann SD et al (2016) Synergistic stress exacerbation in hippocampal neurons: evidence favoring the dual-hit hypothesis of neurodegeneration. Hippocampus 26(8):980–994 26934478 10.1002/hipo.22580 PMC4949116

[CR88] Heller EA, Hamilton PJ (2024) Stereotaxic surgery as a method to deliver epigenetic editing constructs in rodent brain. epigenome editing: methods and protocols. Springer, pp 309–321 10.1007/978-1-0716-4051-7_16 PMC12883188 39012603

[CR89] Hewlings SJ, Kalman DS (2017) Curcumin: a review of its effects on human health. Foods 6(10):92 29065496 10.3390/foods6100092 PMC5664031

[CR90] Höglinger G, Trenkwalder C (2024) Diagnosis and treatment of Parkinson’s disease (guideline of the German Society for Neurology). Neurol Res Pract 6(1):30 38845028 10.1186/s42466-024-00325-4 PMC11157782

[CR91] Hopkins AL (2008) Network pharmacology: the next paradigm in drug discovery. Nat Chem Biol 4(11):682–690 18936753 10.1038/nchembio.118

[CR92] Hu L-W et al (2014) Luteolin modulates 6-hydroxydopamine-induced transcriptional changes of stress response pathways in PC12 cells. PLoS ONE 9(5):e97880 24846311 10.1371/journal.pone.0097880 PMC4028259

[CR93] Hu G et al (2017) Triptolide promotes the clearance of α-synuclein by enhancing autophagy in neuronal cells. Mol Neurobiol 54:2361–2372 26957304 10.1007/s12035-016-9808-3

[CR94] Hua J et al (2017) Ginkgolide B and bilobalide ameliorate neural cell apoptosis in α-synuclein aggregates. Biomed Pharmacother 96:792–797 29054095 10.1016/j.biopha.2017.10.050

[CR95] Huang S et al (2021) Berberine protects against NLRP3 inflammasome via ameliorating autophagic impairment in MPTP-induced Parkinson’s disease model. Front Pharmacol 11:618787 33584302 10.3389/fphar.2020.618787 PMC7872967

[CR96] Huh E et al (2020) Ginger and 6-shogaol protect intestinal tight junction and enteric dopaminergic neurons against 1-methyl-4-phenyl 1, 2, 3, 6-tetrahydropyridine in mice. Nutr Neurosci 23(6):455–464 30230979 10.1080/1028415X.2018.1520477

[CR97] Investigators NN-P (2007) A randomized clinical trial of coenzyme Q10 and GPI-1485 in early Parkinson disease. Neurology 68(1):20–28 17200487 10.1212/01.wnl.0000250355.28474.8e

[CR98] Iqbal MS et al (2024) Progress and trends in neurological disorders research based on deep learning. Compute Med Imaging Graph 116:102400 10.1016/j.compmedimag.2024.102400 38851079

[CR99] Issa MY et al (2020) Neuroprotective effects of Pulicaria undulata essential oil in rotenone model of Parkinson’s disease in rats: insights into its anti-inflammatory and anti-oxidant effects. S Afr J Bot 132:289–298

[CR100] Jadidian F et al (2024) Pharmacotherapeutic potential of Vitis vinifera (grape) in age-related neurological diseases. Boletín Latinoamericano y Del Caribe De Plantas Medicinales y Aromáticas 23(3):349–370

[CR101] Jankovic J, Tan EK (2020) Parkinson’s disease: etiopathogenesis and treatment. J Neurol Neurosurg Psychiatry 91(8):795–808 32576618 10.1136/jnnp-2019-322338

[CR102] Jha SK et al (2015) p38 MAPK and PI3K/AKT signalling cascades in Parkinson’s disease. Int J Mol Cell Med 4(2):67 26261796 PMC4499569

[CR103] Jie Z (2014) Clinical effects and safety of coenzyme Q10 in Parkinson disease. China Foreign Med Treat 23:79–80

[CR104] Jo A et al (2021) PARIS farnesylation prevents neurodegeneration in models of Parkinson’s disease. Science Transl Med 13(604):eaax8891 10.1126/scitranslmed.aax8891 PMC9990146 34321320

[CR105] Joshi N, Singh S (2018) Updates on immunity and inflammation in Parkinson disease pathology. J Neurosci Res 96(3):379–390 29072332 10.1002/jnr.24185

[CR106] Jung UJ, Kim SR (2018) Beneficial effects of flavonoids against Parkinson’s disease. J Med Food 21(5):421–432 29412767 10.1089/jmf.2017.4078

[CR107] Kabuto H, Yamanushi TT (2011) Effects of zingerone [4-(4-hydroxy-3-methoxyphenyl)-2-butanone] and eugenol [2-methoxy-4-(2-propenyl) phenol] on the pathological progress in the 6-hydroxydopamine-induced Parkinson’s disease mouse model. Neurochem Res 36:2244–2249 21769642 10.1007/s11064-011-0548-5

[CR108] Kabuto H et al (2005) Zingerone [4-(4-hydroxy-3-methoxyphenyl)-2-butanone] prevents 6-hydroxydopamine-induced dopamine depression in mouse striatum and increases superoxide scavenging activity in serum. Neurochem Res 30:325–332 16018576 10.1007/s11064-005-2606-3

[CR109] Kabuto H, Tada M, Kohno M (2007) Eugenol [2-methoxy-4-(2-propenyl) phenol] prevents 6-hydroxydopamine-induced dopamine depression and lipid peroxidation inductivity in mouse striatum. Biol Pharm Bull 30(3):423–427 17329831 10.1248/bpb.30.423

[CR110] Kalaba Ö, Güzeloğlu ÖMC (2024) Cognition and quality of life in Parkinson’s disease. Psikiyatride Güncel Yaklaşımlar 16(4):604–616

[CR111] Karuppagounder S et al (2013) Quercetin up-regulates mitochondrial complex-I activity to protect against programmed cell death in rotenone model of Parkinson’s disease in rats. Neuroscience 236:136–148 23357119 10.1016/j.neuroscience.2013.01.032

[CR112] Kasten M et al (2018) Genotype-phenotype relations for the Parkinson’s disease genes Parkin, PINK1, DJ1: MDSGene systematic review. Mov Disord 33(5):730–741 29644727 10.1002/mds.27352

[CR113] Kempuraj D et al (2021) Neuroprotective effects of flavone luteolin in neuroinflammation and neurotrauma. BioFactors 47(2):190–197 33098588 10.1002/biof.1687

[CR114] Kesh S et al (2021) Hesperidin downregulates kinases lrrk2 and gsk3β in a 6-OHDA induced Parkinson’s disease model. Neurosci Lett 740:135426 33075420 10.1016/j.neulet.2020.135426

[CR115] Khani M et al (2024) Towards a global view of Parkinson’s disease genetics. Ann Neurol 95(5):831–842 38557965 10.1002/ana.26905 PMC11060911

[CR116] Khot M et al (2022) NLRP3 inflammasomes: a potential target to improve mitochondrial biogenesis in Parkinson’s disease. Eur J Pharmacol 934:175300 36167151 10.1016/j.ejphar.2022.175300

[CR117] Kim B-W et al (2015) α-Asarone attenuates microglia-mediated neuroinflammation by inhibiting NF kappa B activation and mitigates MPTP-induced behavioral deficits in a mouse model of Parkinson’s disease. Neuropharmacology 97:46–57 25983275 10.1016/j.neuropharm.2015.04.037

[CR118] Kim HD et al (2016) Myricitrin ameliorates 6-hydroxydopamine-induced dopaminergic neuronal loss in the substantia nigra of mouse brain. J Med Food 19(4):374–382 26991235 10.1089/jmf.2015.3581

[CR119] Klemann CJ et al (2017) Integrated molecular landscape of Parkinson’s disease*.* npj Parkinson's Disease **3**(1):14 10.1038/s41531-017-0015-3 PMC5460267 28649614

[CR120] Krishnamoorthy A et al (2019) Chrysin restores MPTP induced neuroinflammation, oxidative stress and neurotrophic factors in an acute Parkinson’s disease mouse model. Neurosci Lett 709:134382 31325581 10.1016/j.neulet.2019.134382

[CR121] Kumar A, Yegla B, Foster TC (2018) Redox signaling in neurotransmission and cognition during aging. Antioxid Redox Signal 28(18):1724–1745 28467718 10.1089/ars.2017.7111 PMC5962336

[CR122] Kumari M et al (2021) Tocotrienols ameliorate neurodegeneration and motor deficits in the 6-OHDA-induced rat model of parkinsonism: behavioural and immunohistochemistry analysis. Nutrients 13(5):1583 34068460 10.3390/nu13051583 PMC8150907

[CR123] Lal R, Chopra K (2024) Experimental models of Parkinson’s disease: challenges and opportunities. Eur J Pharmacol 980:176819 39029778 10.1016/j.ejphar.2024.176819

[CR124] Landucci E et al (2021) Neuroprotective effects of cannabidiol but not Δ9-Tetrahydrocannabinol in rat hippocampal slices exposed to oxygen-glucose deprivation: studies with Cannabis extracts and selected cannabinoids. Int J Mol Sci 22(18):9773 34575932 10.3390/ijms22189773 PMC8468213

[CR125] Langston JW et al (1983) Chronic Parkinsonism in humans due to a product of meperidine-analog synthesis. Science 219(4587):979–980 6823561 10.1126/science.6823561

[CR126] Lashgari N-A et al (2021) The involvement of JAK/STAT signaling pathway in the treatment of Parkinson’s disease. J Neuroimmunol 361:577758 34739911 10.1016/j.jneuroim.2021.577758

[CR127] Lawana V, Cannon JR (2020) *Rotenone neurotoxicity: relevance to Parkinson*’*s disease*. Advances in neurotoxicology. Elsevier, pp 209–254

[CR128] Lee E et al (2014) Baicalein attenuates astroglial activation in the 1-methyl-4-phenyl-1, 2, 3, 4-tetrahydropyridine-induced Parkinson’s disease model by downregulating the activations of nuclear factor-κB, ERK, and JNK. J Neurosci Res 92(1):130–139 24166733 10.1002/jnr.23307

[CR129] Li Z et al (2015) The effect of creatine and coenzyme q10 combination therapy on mild cognitive impairment in Parkinson’s disease. Eur Neurol 73(3–4):205–211 25792086 10.1159/000377676

[CR130] Li X et al (2020a) The critical role of SIRT1 in Parkinson’s disease: mechanism and therapeutic considerations. Aging Dis 11(6):1608 33269110 10.14336/AD.2020.0216 PMC7673849

[CR131] Li X et al (2020b) Ferulic acid ameliorates MPP+/MPTP-induced oxidative stress via ERK1/2-dependent Nrf2 activation: translational implications for Parkinson disease treatment. Mol Neurobiol 57:2981–2995 32445087 10.1007/s12035-020-01934-1

[CR132] Li Y et al (2022) Preclinical reserpine models recapitulating motor and non-motor features of Parkinson’s disease: roles of epigenetic upregulation of alpha-synuclein and autophagy impairment. Front Pharmacol 13:944376 36313295 10.3389/fphar.2022.944376 PMC9597253

[CR133] Li C et al (2024) Systemic inflammation and risk of Parkinson’s disease: a prospective cohort study and genetic analysis. Brain Behav Immun 117:447–455 38336023 10.1016/j.bbi.2024.02.013

[CR134] Lieberman A et al (2019) Nicotine bitartrate reduces falls and freezing of gait in Parkinson disease: a reanalysis. Front Neurol 10:424 31133957 10.3389/fneur.2019.00424 PMC6514133

[CR135] Lieu CA et al (2010) A water extract of Mucuna pruriens provides long-term amelioration of parkinsonism with reduced risk for dyskinesias. Parkinsonism Relat Disord 16(7):458–465 20570206 10.1016/j.parkreldis.2010.04.015 PMC2909380

[CR136] Lieu CA et al (2012) The antiparkinsonian and antidyskinetic mechanisms of Mucuna pruriens in the MPTP-treated nonhuman primate. Evidence-Based Complementary Alternative Med 2012(1):840247 10.1155/2012/840247 PMC3445014 22997535

[CR137] Liu J, Liu W, Yang H (2018) Balancing apoptosis and autophagy for Parkinson’s disease therapy: targeting BCL-2. ACS Chem Neurosci 10(2):792–802 10.1021/acschemneuro.8b00356 30400738

[CR138] Lou H et al (2014) Naringenin protects against 6-OHDA-induced neurotoxicity via activation of the Nrf2/ARE signaling pathway. Neuropharmacology 79:380–388 24333330 10.1016/j.neuropharm.2013.11.026

[CR139] Lu Z et al (2006) Structure–activity relationship analysis of antioxidant ability and neuroprotective effect of gallic acid derivatives. Neurochem Int 48(4):263–274 16343693 10.1016/j.neuint.2005.10.010

[CR140] Lu JY et al (2017) The neuroprotective effect of nicotine in Parkinson’s disease models is associated with inhibiting PARP-1 and caspase-3 cleavage. PeerJ 5:e3933 29062606 10.7717/peerj.3933 PMC5651169

[CR141] Lu B et al (2024) The power of many brains: catalyzing neuropsychiatric discovery through open neuroimaging data and large-scale collaboration. Science Bulletin 69:1536 38519398 10.1016/j.scib.2024.03.006

[CR142] Luan Y et al (2018) Chronic caffeine treatment protects against α-synucleinopathy by reestablishing autophagy activity in the mouse striatum. Front Neurosci 12:301 29770111 10.3389/fnins.2018.00301 PMC5942142

[CR143] Luo Q et al (2022) Association of p53 with neurodegeneration in Parkinson’s disease. Parkinson’s Disease 2022. 10.1155/2022/6600944 10.1155/2022/6600944 PMC9117072 35601652

[CR144] Lutz B (2022) Neurobiology of cannabinoid receptor signaling. Dialogues Clin Neurosci 22:207 10.31887/DCNS.2020.22.3/blutz PMC7605026 33162764

[CR145] Mahapatra P (2018) Identification of natural inhibitors of proteins involved in the pathology of Parkinson’s disease [Master's thesis, Central University of Punjab]. Knowledge Repository

[CR146] Mansouri MT et al (2013) Neuroprotective effects of oral gallic acid against oxidative stress induced by 6-hydroxydopamine in rats. Food Chem 138(2–3):1028–1033 23411210 10.1016/j.foodchem.2012.11.022

[CR147] Maristany AJ et al (2024) Psychiatric manifestations of neurological diseases: a narrative review. Cureus 16(7):e64152 39119372 10.7759/cureus.64152 PMC11308735

[CR148] Marsili L, Marconi R, Colosimo C (2017) Treatment strategies in early Parkinson’s disease. Int Rev Neurobiol 132:345–360 28554414 10.1016/bs.irn.2017.01.002

[CR149] Mathur R, Seamon M (2024) CRISPR technology for Parkinson’s disease: recent advancements and ongoing challenges. STEM Fellowship J (0):1–10. 10.17975/sfj-2024-007

[CR150] Mehta N et al (2023) C-reactive protein as the biomarker of choice to monitor the effects of exercise on inflammation in Parkinson’s disease. Front Immunol 14:1178448 37251392 10.3389/fimmu.2023.1178448 PMC10213511

[CR151] Meng H et al (2017) Loss of Parkinson’s disease-associated protein CHCHD2 affects mitochondrial crista structure and destabilizes cytochrome c. Nat Commun 8(1):15500 28589937 10.1038/ncomms15500 PMC5467237

[CR152] Mesarosova L et al (2024) miR-193b-3p/PGC-1α pathway regulates an insulin dependent anti-inflammatory response in Parkinson’s disease. Neurobiol Dis 199:106587 38950713 10.1016/j.nbd.2024.106587

[CR153] Miao Q et al (2022) The neuroprotective effects and transdifferentiation of astrocytes into dopaminergic neurons of Ginkgolide K on Parkinson’ disease mice. J Neuroimmunol 364:577806 35121334 10.1016/j.jneuroim.2022.577806

[CR154] Mitsui J et al (2017) Three-year follow-up of high-dose ubiquinol supplementation in a case of familial multiple system atrophy with compound heterozygous COQ2 mutations. Cerebellum 16:664–672 28150130 10.1007/s12311-017-0846-9 PMC5427137

[CR155] Moreau C et al (2015) Polymorphism of the dopamine transporter type 1 gene modifies the treatment response in Parkinson’s disease. Brain 138(5):1271–1283 25805645 10.1093/brain/awv063 PMC5963414

[CR156] Morshedi D et al (2015) Cuminaldehyde as the major component of Cuminum cyminum, a natural aldehyde with inhibitory effect on alpha-synuclein fibrillation and cytotoxicity. J Food Sci 80(10):H2336–H2345 26351865 10.1111/1750-3841.13016

[CR157] Muleiro Alvarez M et al (2024) A comprehensive approach to Parkinson’s disease: addressing its molecular, clinical, and therapeutic aspects. Int J Mol Sci 25(13):7183 39000288 10.3390/ijms25137183 PMC11241043

[CR158] Müller T et al (2003) Coenzyme Q10 supplementation provides mild symptomatic benefit in patients with Parkinson’s disease. Neurosci Lett 341(3):201–204 12697283 10.1016/s0304-3940(03)00185-x

[CR159] Nagatsu T et al (2019) Human tyrosine hydroxylase in Parkinson’s disease and in related disorders. J Neural Transm 126:397–409 29995172 10.1007/s00702-018-1903-3

[CR160] Nemade D, Subramanian T, Shivkumar V (2021) An update on medical and surgical treatments of Parkinson’s disease. Aging Dis 12(4):1021 34221546 10.14336/AD.2020.1225 PMC8219497

[CR161] Ng C-H et al (2009) Parkin protects against LRRK2 G2019S mutant-induced dopaminergic neurodegeneration in Drosophila. J Neurosci 29(36):11257–11262 19741132 10.1523/JNEUROSCI.2375-09.2009 PMC2771772

[CR162] Ning B et al (2016) β-Asarone inhibits IRE1/XBP1 endoplasmic reticulum stress pathway in 6-OHDA-induced parkinsonian rats. Neurochem Res 41:2097–2101 27097550 10.1007/s11064-016-1922-0

[CR163] Ning B et al (2019) β-Asarone regulates ER stress and autophagy via inhibition of the PERK/CHOP/Bcl-2/Beclin-1 pathway in 6-OHDA-induced parkinsonian rats. Neurochem Res 44:1159–1166 30796752 10.1007/s11064-019-02757-w

[CR164] Oliveri V (2019) Toward the discovery and development of effective modulators of α-synuclein amyloid aggregation. Eur J Med Chem 167:10–36 30743095 10.1016/j.ejmech.2019.01.045

[CR165] Oliveri V et al (2015) Soluble sugar-based quinoline derivatives as new antioxidant modulators of metal-induced amyloid aggregation. Inorg Chem 54(6):2591–2602 25732904 10.1021/ic502713f

[CR166] Orhan H et al (2021) Toxicology of herbal medicines. toxicology for the health and pharmaceutical sciences. CRC Press, pp 189–220

[CR167] Oyanna V, Clarke J (2024) Mechanisms of intestinal pharmacokinetic natural product-drug interactions. Drug Metabolism Reviews (just-accepted):1–51 10.1080/03602532.2024.2386597 PMC11606768 39078118

[CR168] Pan M-T et al (2024) Genetically modified non-human primate models for research on neurodegenerative diseases. Zool Res 45(2):263 38287907 10.24272/j.issn.2095-8137.2023.197 PMC11017080

[CR169] Pandit N, Kulkarni S, Singhvi G (2024) Effect of green tea on human brain health. nutraceutical fruits and foods for neurodegenerative disorders. Elsevier, pp 301–331

[CR170] Pannu A et al (2021) Emerging role of flavonoids as the treatment of depression. Biomolecules 11(12):1825 34944471 10.3390/biom11121825 PMC8698856

[CR171] Panossian AT, Lemerond T, Efferth T (2024) Botanical hybrid preparations (BHP) in phytomedicine and phytotherapy research: background and perspectives. Pharmaceuticals 17(4):483 38675443 10.3390/ph17040483 PMC11053582

[CR172] Park G et al (2013) 6-Shogaol, an active compound of ginger, protects dopaminergic neurons in Parkinson’s disease models via anti-neuroinflammation. Acta Pharmacol Sin 34(9):1131–1139 23811724 10.1038/aps.2013.57 PMC4003157

[CR173] Park HW et al (2020) Intrastriatal administration of coenzyme Q10 enhances neuroprotection in a Parkinson’s disease rat model. Sci Rep 10(1):9572 32533070 10.1038/s41598-020-66493-w PMC7293316

[CR174] Pasban-Aliabadi H et al (2013) Inhibition of 6-hydroxydopamine-induced PC12 cell apoptosis by olive (Olea europaea L.) leaf extract is performed by its main component oleuropein. Rejuvenation Res 16(2):134–142 23394606 10.1089/rej.2012.1384

[CR175] Pinna A (2014) Adenosine A2A receptor antagonists in Parkinson’s disease: progress in clinical trials from the newly approved istradefylline to drugs in early development and those already discontinued. CNS Drugs 28(5):455–474 24687255 10.1007/s40263-014-0161-7

[CR176] Postuma RB et al (2017) Caffeine as symptomatic treatment for Parkinson disease (Café-PD): a randomized trial. Neurology 89(17):1795–1803 28954882 10.1212/WNL.0000000000004568 PMC5664303

[CR177] Prasad K et al (2024) Effects of the adenosine A2A receptor antagonist KW6002 on the dopaminergic system, motor performance, and neuroinflammation in a rat model of Parkinson’s disease. Neuropharmacology 247:109862 38325770 10.1016/j.neuropharm.2024.109862

[CR178] Prymaczok NC et al (2024) Cell-to-cell transmitted alpha-synuclein recapitulates experimental Parkinson’s disease. npj Parkinson’s Dis 10(1):10 38184623 10.1038/s41531-023-00618-6 PMC10771530

[CR179] Qualls Z et al (2014) Protective effects of curcumin against rotenone and salsolinol-induced toxicity: implications for Parkinson’s disease. Neurotox Res 25:81–89 24122264 10.1007/s12640-013-9433-0 PMC3945160

[CR180] Quik M et al (2015) Alpha7 nicotinic receptors as therapeutic targets for Parkinson’s disease. Biochem Pharmacol 97(4):399–407 26093062 10.1016/j.bcp.2015.06.014 PMC4600450

[CR181] Rabey J et al (1993) Broad bean (Vicia faba) consumption and Parkinson’s disease. Adv Neurol 60:681–684 8420210

[CR182] Rai SN, Singh P (2020) Advancement in the modelling and therapeutics of Parkinson’s disease. J Chem Neuroanat 104:101752 31996329 10.1016/j.jchemneu.2020.101752

[CR183] Ramazani E et al (2020) Protective effects of Cinnamomum verum, Cinnamomum cassia and cinnamaldehyde against 6-OHDA-induced apoptosis in PC12 cells. Mol Biol Rep 47:2437–2445 32166553 10.1007/s11033-020-05284-y

[CR184] Ramirez AI et al (2017) The role of microglia in retinal neurodegeneration: Alzheimer’s disease, Parkinson, and glaucoma. Frontiers in Aging Neuroscience 9:214 28729832 10.3389/fnagi.2017.00214 PMC5498525

[CR185] Rao AS et al (2024) Impacts of omega-3 fatty acids, natural elixirs for neuronal health, on brain development and functions. neuroprotection: method and protocols. Springer, pp 209–229 10.1007/978-1-0716-3662-6_15 38427239

[CR186] Rashed AA, Rahman AZA, Rathi DNG (2021) Essential oils as a potential neuroprotective remedy for age-related neurodegenerative diseases: a review. Molecules 26(4):1107 33669787 10.3390/molecules26041107 PMC7922935

[CR187] Rétey J et al (2007) A genetic variation in the adenosine A2A receptor gene (ADORA2A) contributes to individual sensitivity to caffeine effects on sleep. Clin Pharmacol Ther 81(5):692–698 17329997 10.1038/sj.clpt.6100102

[CR188] Ricke KM et al (2020) Mitochondrial dysfunction combined with high calcium load leads to impaired antioxidant defense underlying the selective loss of nigral dopaminergic neurons. J Neurosci 40(9):1975–1986 32005765 10.1523/JNEUROSCI.1345-19.2019 PMC7046450

[CR189] Rieck M et al (2015) Is there a role for ADORA2A polymorphisms in levodopa-induced dyskinesia in Parkinson’s disease patients? Pharmacogenomics 16(6):573–582 25872644 10.2217/pgs.15.23

[CR190] Rijntjes M (2019) Knowing your beans in Parkinson’s disease: a critical assessment of current knowledge about different beans and their compounds in the treatment of Parkinson’s disease and in animal models. Parkinson’s Disease 2019(1):1349509 31781363 10.1155/2019/1349509 PMC6875167

[CR191] Rinwa P, Kumar A (2017) Quercetin along with piperine prevents cognitive dysfunction, oxidative stress and neuro-inflammation associated with mouse model of chronic unpredictable stress. Arch Pharmacal Res 40:1166–1175 10.1007/s12272-013-0205-4 23856969

[CR192] Rosado-Ramos R et al (2021) Small molecule fisetin modulates alpha–synuclein aggregation. Molecules 26(11):3353 34199487 10.3390/molecules26113353 PMC8199635

[CR193] Rui W et al (2020) Baicalein attenuates neuroinflammation by inhibiting NLRP3/caspase-1/GSDMD pathway in MPTP-induced mice model of Parkinson’s disease. Int J Neuropsychopharmacol 23(11):762–773 32761175 10.1093/ijnp/pyaa060 PMC7745250

[CR194] Ruszkiewicz J, Albrecht J (2015) Changes in the mitochondrial antioxidant systems in neurodegenerative diseases and acute brain disorders. Neurochem Int 88:66–72 25576182 10.1016/j.neuint.2014.12.012

[CR195] Sadlon AE, Lamson DW (2010) Immune-modifying and antimicrobial effects of eucalyptus oil and simple inhalation devices. Altern Med Rev 15(1):33–43 20359267

[CR196] Scarpulla RC (2011) Metabolic control of mitochondrial biogenesis through the PGC-1 family regulatory network*.* Biochimica et Biophysica Acta (BBA)-Mol Cell Res 1813(7):1269–1278 10.1016/j.bbamcr.2010.09.019 PMC3035754 20933024

[CR197] Scherma M et al (2016) Interactions between the endocannabinoid and nicotinic cholinergic systems: preclinical evidence and therapeutic perspectives. Psychopharmacology 233:1765–1777 26728894 10.1007/s00213-015-4196-3

[CR198] Sedaghat R, Roghani M, Khalili M (2014) Neuroprotective effect of thymoquinone, the nigella sativa bioactive compound, in 6-hydroxydopamine-induced hemi-parkinsonian rat model. Iranian J Pharmaceutical Res: IJPR 13(1):227 PMC3985249 24734075

[CR199] Shahpiri Z et al (2016) Phytochemicals as future drugs for Parkinson’s disease: a comprehensive review. Rev Neurosci 27(6):651–668 27124673 10.1515/revneuro-2016-0004

[CR200] Shin J-H et al (2011) PARIS (ZNF746) repression of PGC-1α contributes to neurodegeneration in Parkinson’s disease. Cell 144(5):689–702 21376232 10.1016/j.cell.2011.02.010 PMC3063894

[CR201] Shrivastava P et al (2013) Anti-apoptotic and anti-inflammatory effect of piperine on 6-OHDA induced Parkinson’s rat model. J Nutr Biochem 24(4):680–687 22819561 10.1016/j.jnutbio.2012.03.018

[CR202] Shults CW et al (2002) Effects of coenzyme Q10 in early Parkinson disease: evidence of slowing of the functional decline. Arch Neurol 59(10):1541–1550 12374491 10.1001/archneur.59.10.1541

[CR203] Sidransky E et al (2009) *Multicenter analysis of glucocerebrosidase mutations in Parkinson*’*s disease*. N Engl J Med 361(17):1651–1661 19846850 10.1056/NEJMoa0901281 PMC2856322

[CR204] Skou LD et al (2024) Pathogenesis of DJ-1/PARK7-mediated Parkinson’s disease. Cells 13(4):296 38391909 10.3390/cells13040296 PMC10887164

[CR205] Smith L, Schapira AH (2022) GBA variants and Parkinson disease: mechanisms and treatments. Cells 11(8):1261 35455941 10.3390/cells11081261 PMC9029385

[CR206] So Y-J et al (2024) The potentiality of natural products and herbal medicine as novel medications for Parkinson’s disease: a promising therapeutic approach. Int J Mol Sci 25(2):1071 38256144 10.3390/ijms25021071 PMC10816678

[CR207] Solayman M et al (2017) Natural products combating neurodegeneration: Parkinson’s disease. Curr Drug Metab 18(1):50–61 27396919 10.2174/1389200217666160709204826

[CR208] Soner BC et al (2021) Neuroprotective effect of intrastriatal caffeic acid phenethyl ester treatment in 6-OH dopamine model of Parkinson’s disease in rats. Parkinson’s Disease 2021:1 10.1155/2021/5553480 PMC8424247 34512945

[CR209] Song Q, Peng S, Zhu X (2021) Baicalein protects against MPP+/MPTP-induced neurotoxicity by ameliorating oxidative stress in SH-SY5Y cells and mouse model of Parkinson’s disease. Neurotoxicology 87:188–194 34666128 10.1016/j.neuro.2021.10.003

[CR210] Speciale SG (2002) MPTP: insights into parkinsonian neurodegeneration. Neurotoxicol Teratol 24(5):607–620 12200192 10.1016/s0892-0362(02)00222-2

[CR211] Stamelou M et al (2008) Short-term effects of coenzyme Q10 in progressive supranuclear palsy: a randomized, placebo-controlled trial. Move Disord: Off J Move Disord Soc 23(7):942–949 10.1002/mds.22023 18464278

[CR212] Stanford SC, Heal DJ (2019) Catecholamines: knowledge and understanding in the 1960s, now, and in the future. Brain Neurosci Adv 3:2398212818810682 32166174 10.1177/2398212818810682 PMC7058270

[CR213] Storch A et al (2007) Randomized, double-blind, placebo-controlled trial on symptomatic effects of coenzyme Q10 in Parkinson disease. Arch Neurol 64(7):938–944 17502459 10.1001/archneur.64.7.nct60005

[CR214] Strijks E, Kremer H, Horstink M (1997) Q10 therapy in patients with idiopathic Parkinson’s disease. Mol Aspects Med 18:237–240 10.1016/s0098-2997(97)00008-3 9266528

[CR215] Subramaniam SR, Ellis EM (2013) *Neuroprotective effects of umbelliferone and esculetin in a mouse model of Parkinson*’*s disease*. J Neurosci Res 91(3):453–461 23184853 10.1002/jnr.23164

[CR216] Sun Y et al (2021) Therapeutic opportunities of interleukin-33 in the central nervous system. Front Immunol 12:654626 34079543 10.3389/fimmu.2021.654626 PMC8165230

[CR217] Tamilselvam K et al (2013) Neuroprotective effects of hesperidin, a plant flavanone, on rotenone-induced oxidative stress and apoptosis in a cellular model for Parkinson’s disease. Oxidative Med Cell Longevity 2013:1 10.1155/2013/102741 PMC3800605 24205431

[CR218] Tan Y-Y, Jenner P, Chen S-D (2022) Monoamine oxidase-B inhibitors for the treatment of Parkinson’s disease: past, present, and future. J Parkinsons Dis 12(2):477–493 34957948 10.3233/JPD-212976 PMC8925102

[CR219] Tanriover G et al (2010) The effects of docosahexaenoic acid on glial derived neurotrophic factor and neurturin in bilateral rat model of Parkinson’s disease. Folia Histochem Cytobiol 48(3):434–441 21071351 10.2478/v10042-010-0047-6

[CR220] Tavassoly O et al (2014) The use of nanopore analysis for discovering drugs which bind to α-synuclein for treatment of Parkinson’s disease. Eur J Med Chem 88:42–54 25081642 10.1016/j.ejmech.2014.07.090

[CR221] Taylor JM, Main BS, Crack PJ (2013) Neuroinflammation and oxidative stress: co-conspirators in the pathology of Parkinson’s disease. Neurochem Int 62(5):803–819 23291248 10.1016/j.neuint.2012.12.016

[CR222] Thangavelu L et al (2024) Non-coding RNAs in Parkinson’s disease: regulating SNCA and alpha-synuclein aggregation. Pathology-Res Pract 261:155511 10.1016/j.prp.2024.155511 39094523

[CR223] Tharakan B et al (2007) Anti-Parkinson botanical Mucuna pruriens prevents levodopa induced plasmid and genomic DNA damage. Phytotherapy Res: Int J Devoted Pharmacol Toxicol Evaluation Nat Product Derivatives 21(12):1124–1126 10.1002/ptr.2219 17622977

[CR224] Thirugnanam T, Santhakumar K (2022) Chemically induced models of Parkinson’s disease. Comp Biochem Physiol c: Toxicol Pharmacol 252:109213 34673252 10.1016/j.cbpc.2021.109213

[CR225] Trist BG, Hare DJ, Double KL (2019) Oxidative stress in the aging substantia nigra and the etiology of Parkinson’s disease. Aging Cell 18(6):e13031 31432604 10.1111/acel.13031 PMC6826160

[CR226] Tufekci KU, Genc S, Genc K (2011) The Endotoxin-induced neuroinflammation model of Parkinson’s disease. Parkinson’s Disease 2011(1):487450 21331154 10.4061/2011/487450 PMC3034925

[CR227] Turan D et al (2020) Evaluation of the neuroprotective potential of caffeic acid phenethyl ester in a cellular model of Parkinson’s disease. Eur J Pharmacol 883:173342 32634439 10.1016/j.ejphar.2020.173342

[CR228] Turer BY Sanlier N (2024) Relationship of curcumin with aging and Alzheimer and Parkinson disease, the most prevalent age-related neurodegenerative diseases: a narrative review*.* Nutrition Rev nuae079. 10.1093/nutrit/nuae079 10.1093/nutrit/nuae079 38916925

[CR229] Ungerstedt U (1968) 6-Hydroxy-dopamine induced degeneration of central monoamine neurons. Eur J Pharmacol 5(1):107–110 5718510 10.1016/0014-2999(68)90164-7

[CR230] Vijayakumar S et al (2016) Review on potential phytocompounds in drug development for Parkinson disease: a pharmacoinformatic approach. Inform Med Unlocked 5:15–25

[CR231] Wang X et al (2014) Clinical observation of coenzyme Q10 in Parkinson disease. HeBei J TCM 36:151–153

[CR232] Wang S et al (2015) Tanshinone I selectively suppresses pro-inflammatory genes expression in activated microglia and prevents nigrostriatal dopaminergic neurodegeneration in a mouse model of Parkinson’s disease. J Ethnopharmacol 164:247–255 25666429 10.1016/j.jep.2015.01.042

[CR233] Wang ZY et al (2017a) Neuroprotective natural products for the treatment of Parkinson’s disease by targeting the autophagy–lysosome pathway: a systematic review. Phytother Res 31(8):1119–1127 28504367 10.1002/ptr.5834

[CR234] Wang H et al (2017b) Protective effect of naringin against the LPS-induced apoptosis of PC12 cells: implications for the treatment of neurodegenerative disorders. Int J Mol Med 39(4):819–830 28260042 10.3892/ijmm.2017.2904 PMC5360435

[CR235] Wang T et al (2020) Neuroprotective effects of Danshensu on rotenone-induced Parkinson’s disease models in vitro and in vivo. BMC Complementary Med Therapies 20(1):1–10 10.1186/s12906-019-2738-7 PMC7076814 32020857

[CR236] Wang L et al (2021a) Treatment of Parkinson’s disease in zebrafish model with a berberine derivative capable of crossing blood brain barrier, targeting mitochondria, and convenient for bioimaging experiments. Comp Biochem Physiol c: Toxicol Pharmacol 249:109151 34343700 10.1016/j.cbpc.2021.109151

[CR237] Wang W-W et al (2021b) Administration of quercetin improves mitochondria quality control and protects the neurons in 6-OHDA-lesioned Parkinson’s disease models. Aging (Albany NY) 13(8):11738 33878030 10.18632/aging.202868 PMC8109056

[CR238] Williamson EM (2003) Drug interactions between herbal and prescription medicines. Drug Saf 26:1075–1092 14640772 10.2165/00002018-200326150-00002

[CR239] Wilson V, Maulik SK (2018) Herb-drug interactions in neurological disorders: a critical appraisal. Curr Drug Metab 19(5):443–453 29086684 10.2174/1389200218666171031123738

[CR240] Wu C-R et al (2015) Carnosic acid protects against 6-hydroxydopamine-induced neurotoxicity in in vivo and in vitro model of Parkinson’s disease: involvement of antioxidative enzymes induction. Chem Biol Interact 225:40–46 25446857 10.1016/j.cbi.2014.11.011

[CR241] Wu T et al (2020) Synergistic effects of ginkgolide B and protocatechuic acid on the treatment of Parkinson’s disease. Molecules 25(17):3976 32878312 10.3390/molecules25173976 PMC7504731

[CR242] Xu C-L et al (2013) Neuroprotective effects of madecassoside in early stage of Parkinson’s disease induced by MPTP in rats. Fitoterapia 90:112–118 23876367 10.1016/j.fitote.2013.07.009

[CR243] Xu J et al (2017) Resolvin D1 attenuates Mpp+-induced Parkinson disease via inhibiting inflammation in PC12 cells. Med Sci Monit: Int Med J Expt Clin Res 23:2684 10.12659/MSM.901995 PMC5465971 28572562

[CR244] Xu Z et al (2021) Astragaloside IV protects 6-hydroxydopamine-induced SH-SY5Y cell model of Parkinson’s disease via activating the JAK2/STAT3 pathway. Front Neurosci 15:631501 33833662 10.3389/fnins.2021.631501 PMC8021720

[CR245] Yadav SK, Rai SN, Singh SP (2017) Mucuna pruriens reduces inducible nitric oxide synthase expression in Parkinsonian mice model. J Chem Neuroanat 80:1–10 27919828 10.1016/j.jchemneu.2016.11.009

[CR246] Yan X et al (2017) Vanillin protects dopaminergic neurons against inflammation-mediated cell death by inhibiting ERK1/2, P38 and the NF-κB signaling pathway. Int J Mol Sci 18(2):389 28208679 10.3390/ijms18020389 PMC5343924

[CR247] Yang W et al (2015) Neuroprotective effects of piperine on the 1-methyl-4-phenyl-1, 2, 3, 6-tetrahydropyridine-induced Parkinson’s disease mouse model. Int J Mol Med 36(5):1369–1376 26648012 10.3892/ijmm.2015.2356

[CR248] Ye D et al (2024) Adeno-associated virus vector delivery to the brain: technology advancements and clinical applications. Adv Drug Delivery Rev 211:115363 10.1016/j.addr.2024.115363 PMC11892011 38906479

[CR249] Yergök RA et al (2024) Brain-targeted nano-drug delivery for the treatment of Parkinson’s disease. Curr Res Health Sci 1(2):77–92

[CR250] Yoritaka A et al (2015) Randomized, double-blind, placebo-controlled pilot trial of reduced coenzyme Q10 for Parkinson’s disease. Parkinsonism Relat Disord 21(8):911–916 26054881 10.1016/j.parkreldis.2015.05.022

[CR251] Zhang S et al (2020) Anti-Parkinson’s disease activity of phenolic acids from Eucommia ulmoides Oliver leaf extracts and their autophagy activation mechanism. Food Funct 11(2):1425–1440 31971191 10.1039/c9fo02288k

[CR252] Zhou T, Zhu M, Liang Z (2018) (-)-Epigallocatechin-3-gallate modulates peripheral immunity in the MPTP-induced mouse model of Parkinson’s disease. Mol Med Rep 17(4):4883–4888 29363729 10.3892/mmr.2018.8470 PMC5865947

